# Mechanisms of local regeneration and community assembly under broad-leaved Korean pine (*Pinus koraiensis*) forests in northeastern China

**DOI:** 10.7717/peerj.21819

**Published:** 2026-11-04

**Authors:** Xin Du, Shuli He, Benkuan Dong, Yelin Zhang, Huiwu Jiang

**Affiliations:** 1 Basic Teaching and Research Department, East University of Heilongjiang, Harbin, Heilongjiang Province, China; 2 College of Forestry, Northeast Forestry University, Harbin, Heilongjiang Province, China; 3 Faculty Development Center, East University of Heilongjiang, Harbin, Heilongjiang Province, China; 4 Heilongiiang Forestry and Grassland Survey and Planning Design Institute, Harbin, Heilongjiang Province, China

**Keywords:** Broad-leaved Korean pine forests, Seedling, Sapling, Local regeneration community, Community assembly, Canopy environment, Trait

## Abstract

**Aims:**

Seedling and sapling community assembly is a key process affecting forest regeneration and succession, yet the spatial aggregation patterns and assembly mechanisms of locally regenerating communities in broad-leaved Korean pine (*Pinus koraiensis*) forests remain poorly understood. To address this gap, we identified aggregation scales and patch sizes and elucidated the assembly mechanisms of local seedling and sapling communities.

**Methods:**

We conducted stand and regeneration surveys in four broad-leaved Korean pine forest plots. For each regenerating individual, we recorded its species identity, spatial position, and height. Leaf traits were measured at the species level, and local canopy environmental variables were quantified based on stand survey data. Ripley’s *g(r)*, fitted using a Thomas process with a two-nested-scale function, was used to identify aggregation scales, and hierarchical clustering was used to delineate local communities. Abundance-based and presence-based standardized effect sizes of mean pairwise trait distance (SES.MPD), standardized effect sizes of Pielou’s evenness (SES.E), and the degree of distribution tendency (*D*) were then used to characterize community structure, and redundancy analysis (RDA) was used to assess trait–environment relationships.

**Results:**

Both local seedling and sapling communities exhibited two characteristic aggregation scales, at approximately 1 and 15 m. Compared with sapling communities, seedling communities showed stronger aggregation at both the small and large scales, had more patches and more individuals per patch. Across 1–15 m scales, abundance-weighted *SES.MPD*, presence-based *SES.MPD*, *SES.E*, and their corresponding *D* values indicated non-random functional structure in both communities, with stronger abundance-based patterns in seedlings but more consistent presence-based patterns in saplings, and stronger signals of competition at smaller scales in saplings. The RDA showed that environmental explanatory power was relatively weak overall, but increased with spatial scale and developmental stage. Compared with the seedling communities, the sapling communities demonstrated weaker or even opposite correlations in community-weighted mean leaf area (CWM.LA), specific leaf area (SLA), and leaf width (LW) with vertical distribution tendency degrees and local coniferous leaf area index.

**Conclusions:**

Overall, stochastic processes played a major role in shaping local regeneration communities. Limited dispersal caused regenerating individuals with similar traits to cluster across spatial scales. As seedlings developed into saplings, the influence of canopy conditions progressively accumulated, and local competition apparently became stronger, especially at small spatial scales. These findings suggest that the mechanisms shaping regeneration communities change during development, and that seedlings and saplings respond differently to canopy structure.

## Introduction

Natural regeneration is one of the key processes underlying forest renewal, being defined as the replacement of old trees by a new generation of seedlings and saplings (Du et al., 2024; Sundriyal, Joshi & Negi, 2025), and plays a crucial role in ecosystem recovery and succession by shaping future species composition, stand structure, and biodiversity (Siminski et al., 2021; Elsy et al., 2023; Liu, 2025). Therefore, mechanisms of natural regeneration have received broad attention in forest ecology worldwide. Previous studies have shown that natural regeneration in different forest types is jointly regulated by multiple mechanisms, although the dominant factors may vary with forest type and environmental context. For example, in temperate mixed and old-growth forests, gap formation, understory light availability, microtopography, soil conditions, and neighborhood competition are often considered important factors shaping the spatial patterns of seedlings and saplings (Emborg, 1998; Gratzer & Waagepetersen, 2018; Jaloviar et al., 2020). In boreal coniferous forests, seedbed conditions, moss or litter cover, soil disturbance, and overstory structure play important roles in determining regeneration success (Drössler et al., 2017; Mallik & Kayes, 2018; Rautio et al., 2023). By contrast, in species-rich tropical forests, when coexisting tree species are relatively similar in life form and resource-use strategies, propagule-supply processes, such as seed rain, propagule arrival and arrival order, and the occupation of available establishment sites, may also influence seedling regeneration and community composition, indicating that these patterns are not determined solely by environmental conditions or neighborhood relationships (Chave, 2004; Hubbell, 2005; Myers & Harms, 2009). These studies indicate that natural regeneration is shaped by multiple ecological processes, with their relative importance varying across forest types, spatial scales, and developmental stages.

The ecological mechanisms influencing regeneration and growth can be categorized into stochastic and deterministic processes (Liu, Chen & Yang, 2018; Sonnier, Johnson & Waller, 2020). Stochastic processes, as conceptualized by neutral theory (Chave, 2004; Hubbell, 2005), assume ecological equivalence among species and emphasize random demographic events in community assembly. In the context of understory regeneration, stochasticity implies comparable access of propagules to understory microsites, with limited niche differentiation. Deterministic processes, in contrast, encompass dispersal limitation, environmental filtering, and intraspecific and interspecific interactions, highlighting the role of environmental-biotic interactions. For example, dispersal limitation suggests that the distance between suitable habitat patches and parent trees affects establishment (Zhang et al., 2020). Moreover, spatial and temporal heterogeneity in understory conditions—such as light availability, temperature, moisture, soil nutrients, and litter—strongly influences regeneration and growth (Dusan et al., 2007; Čater, Diaci & Roženbergar, 2014; Liu et al., 2020; Liang & Wei, 2020; Hogan et al., 2022; Zhao, Sun & Gao, 2023; Yao et al., 2024; Liu, 2025). Furthermore, the abundance of understory shrubs, herbs, and existing trees can affect the success rate of subsequent regeneration (Thrippleton et al., 2016; Wang et al., 2024b). Such effects are closely linked to functional variation and phylogenetic relationships (Yan et al., 2012; Wang et al., 2024a). Additionally, environmental effects may vary across developmental stages (Abdullah & Sun, 2017; Petrokas, Manton & Kavaliauskas, 2024).

Both deterministic and stochastic mechanisms are scale dependent, and understory communities are shaped by their combined influence, with their relative contributions varying across spatial and temporal scales (Ning et al., 2019). This scale dependence is also reflected in the fact that different ecological processes often operate over different spatial ranges. For example, rodent-mediated dispersal of *Pinus koraiensis* seeds generally occurs within a range of approximately 0–22 m (Yan et al., 2022); the competitive effects between Douglas-fir and understory shrubs have been reported to operate at a scale of about 2.1 m (Wagner & Radosevich, 1991); and neighborhood interference from overstory trees may occur within approximately 5–6 m (Guo & Yang, 2006). Many point-pattern analyses have also shown that the spatial distribution of understory regeneration and its association with parent trees may vary across aggregation scales (Liu, Li & Jin, 2014; Dong et al., 2020). In addition, seed dispersal can promote conspecific aggregation around parent trees, whereas density-dependent effects may weaken recruitment in the immediate neighborhood of adult trees. Together, these processes may generate ring-like aggregation patterns around parent trees, indicating that the mechanisms shaping regeneration communities can vary across spatial scales (Wang et al., 2019).

In climatic climax forest communities (Clements, 1936), the spatial distribution, species composition, and functional trait patterns of understory natural regeneration reflect the integrated outcome of multi-species co-evolution (Chai et al., 2016; Wang et al., 2024a, 2024b; Du et al., 2025a), thereby providing a theoretical basis for forest management and ecological restoration. The broad-leaved Korean pine (*Pinus koraiensis*) forest, as the zonal climax vegetation in the mountainous regions of Northeast Asia, has long been a focus of extensive research on its regeneration mechanisms, yielding a substantial body of literature (Du et al., 2024, 2025a; Jin et al., 2007a, 2007b; Guo, 2002; Liu, Jin & Li, 2014; Zang, Guo & Gao, 1998). However, in the aforementioned studies, most research on tree regeneration have focused on the species level, whereas community-level research has been primarily conducted at the stand scale. In contrast, research on the spatial heterogeneity and patch mosaic patterns of understory regeneration remains relatively limited. As a typical uneven-aged, multi-layered ecosystem with a patch-mosaic structure, the spatial pattern of the broad-leaved Korean pine forest is primarily shaped by local-scale successional processes driven by canopy gap disturbances (Xu, 2001; Zang, Liu & Dong, 1999). Canopy gaps are formed when overstory trees age and die. In the early stages following gap formation, species with strong dispersal ability and rapid growth rates can quickly occupy the vacant niches within the gaps. The formation of canopy gaps and growth of these early colonizers can significantly alter the local understory environmental conditions, including factors such as light availability, temperature, moisture, soil nutrient status, and microbial activity, which in turn influence the establishment and development of slower-growing or later-arriving species. As these initially dominant species grow vertically and the crowns of surrounding gap-edge trees gradually expand inward, the canopy gap begins to close. Compared with closed-canopy understory habitats, the higher radiation flux, temperature, and soil water availability in canopy gaps may promote organic matter decomposition and mineralization, thereby increasing soil nutrient availability (Xu, 2001). As the canopy gap gradually closes, radiation flux decreases, and soil processes also shift: humification may be enhanced, leading to increases in soil organic matter, humic acid content, microbial biomass, and acid phosphatase activity, whereas mineralization tends to weaken (Muscolo, Sidari & Mercurio, 2007; Worrall, Lee & Harrington, 2005). During this process, more shade-tolerant and longer-lived species are better able to endure the environmental filtering exerted by the overstory and thus gradually replace the early-successional species and become the dominant species within the local patch (Zang, Guo & Gao, 1998; Zang, Liu & Dong, 1999). In this process, ecological mechanisms such as environmental filtering, dispersal limitation, interspecific interactions, the Janzen-Connell effect, and limiting similarity may operate jointly across spatial scales. Meanwhile, species’ habitat preferences, resource-use spectra, and survival strategies are closely linked to their functional traits. At the local scale, understory regeneration plant clusters shaped by propagule input (dispersal), environmental filtering, and intra- and interspecific interactions can be viewed as fundamental units of the patch-mosaic structure of the regional regeneration community—*i.e*., “elementary components of community-level differentiation” (Wu, 2007; Leibold & Chase, 2018). From a metacommunity perspective, these local units act as nodes that collectively constitute the regional community and are connected *via* propagule dispersal (Leibold & Chase, 2018). For tree regeneration, local regeneration communities function primarily as receivers, with propagule sources derived from parent trees and the soil seed bank; therefore, local regeneration communities can be viewed as the sink component in a self-organizing source-sink metacommunity framework, providing a basis for discussing the joint effects of dispersal inputs and canopy-mediated environmental filtering on regeneration assembly (Cornacchia et al., 2018; Mouquet & Loreau, 2003).

The local regeneration communities in the understory of broad-leaved Korean pine forests are a concrete manifestation of natural regeneration processes at a microscale and thus hold unique value for exploring the mechanisms of forest succession and community assembly. However, the spatial heterogeneity and patchy mosaic structure of understory regeneration communities in broadleaved Korean pine forests remain poorly understood. Accordingly, this study investigates the spatial patterns and assembly mechanisms of naturally regenerated local communities. Based on previous studies, we propose three hypotheses: (1) Due to limited seed dispersal and microhabitat heterogeneity, understory regeneration communities exhibit significant spatial aggregation at local scales. (2) Community structure is jointly shaped by dispersal limitation and environmental filtering, while limiting similarity further constrains species coexistence, and the relative importance of these processes varies across aggregation scales. (3) Variations in canopy structure significantly influence the establishment success of understory regeneration species and result in functional trait differentiation at the community level. To test these hypotheses, we integrated field surveys with functional trait measurements and applied two R packages independently developed by our team, *M.COMMUNITIES* (https://github.com/DuXinChina/M.COMMUNITIES) and *Broadleaf.Korean.pine.LAI* (https://github.com/DuXinChina/Broadleaf.Korean.pine.LAI), as the main analytical tools (Du et al., 2025a, 2025b). The overall workflow of the study is presented in Fig. S1. Specifically, the objectives were to: (1) To quantify the spatial aggregation scales and intensities of local regeneration communities in the understory; (2) To assess how dispersal limitation, environmental filtering, and limiting similarity jointly shape community assembly across different spatial scales; (3) To elucidate how local canopy environmental factors influence the species composition of understory regeneration communities through the selection of specific functional traits.

## Materials and Methods

### Study area

The study area was located in the Liangshui National Nature Reserve (128°53′20″E, 47°10′50″N), Yichun City, Heilongjiang Province, China. The area lies within the Liangshuigou watershed, situated on the eastern slope of the Dali Dailing branch along the southern slope of the Lesser Khingan Mountains. This region belongs to the temperate coniferous–broadleaved mixed forest zone and features typical low mountainous and hilly terrain, with broadleaved Korean pine forest serving as the zonal climax vegetation type. Figure 1 shows the location of the study area and the appearance of the forest stand.

**Figure 1:**
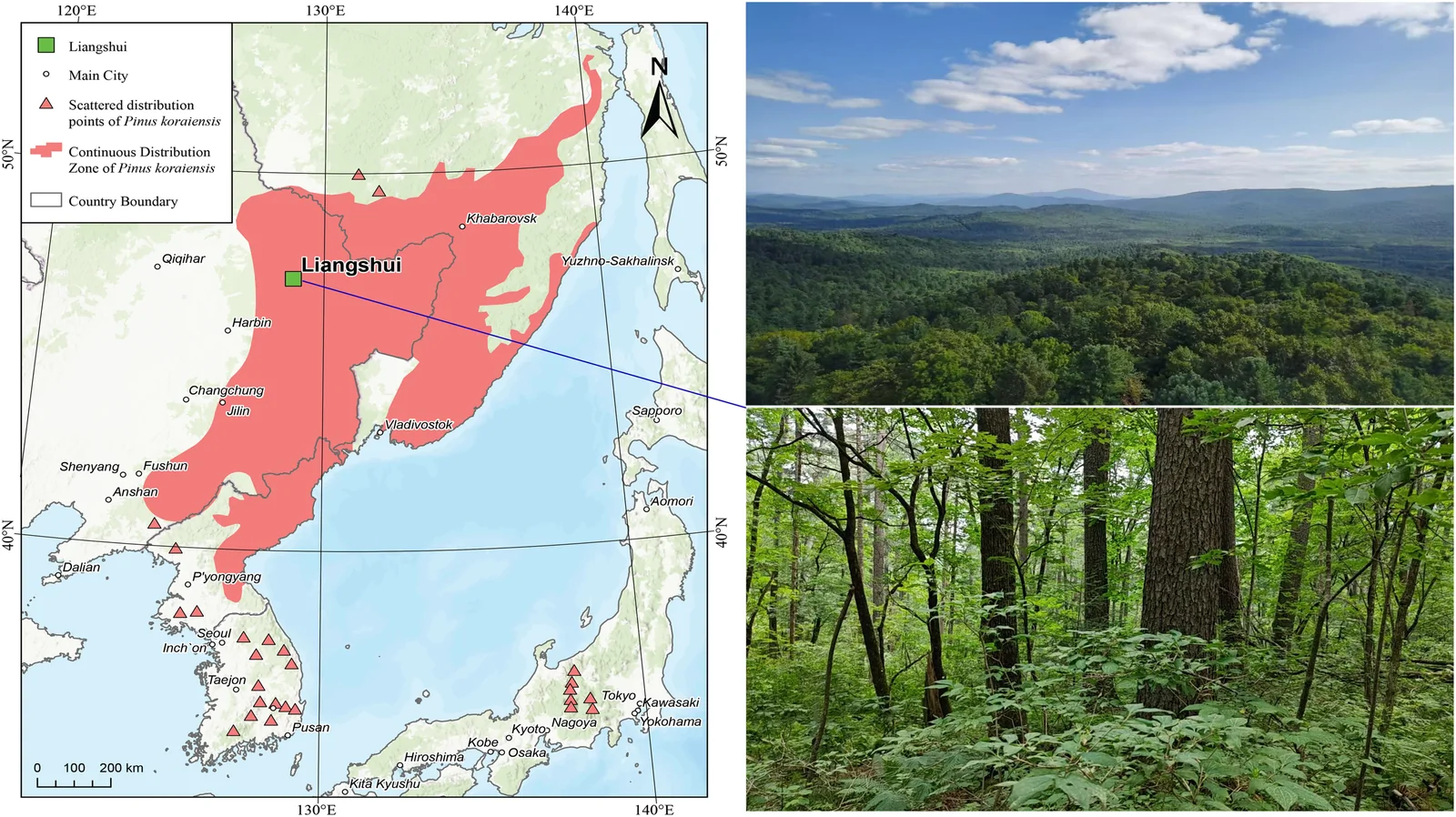
Location map of the study area. The delimitation of the distribution zone and distribution points refers to Xu (2001).

The study area has a temperate continental monsoon climate characterized by cold, dry winters and warm, rainy summers. The mean annual temperature is −0.3 °C, with an average maximum of 7.5 °C and a minimum of −6.6 °C. The average annual precipitation is approximately 676 mm, with the majority occurring between June and August. Snow cover lasts for approximately 130–150 days annually, while the frost-free period ranges from 100 to 120 days. The annual accumulated temperature (above 0 °C) ranges from 2,200 °C to 2,600 °C. The zonal soil type in the study area is dark brown soil, accompanied by intrazonal soils such as meadow soil, gley soil, peat soil, and albic soil. Influenced by slope position and soil moisture conditions, soils in lower slope positions and valley bottoms often show gleying characteristics, whereas soils at higher slope positions gradually transition to typical dark brown forest soils. Because precipitation is relatively abundant in the study area, humus derived from the decomposition of *Pinus koraiensis* needles and other forest litter accumulates in the understory soil, accompanied by leaching and clayification processes. As a result, the understory soils are generally weakly acidic.

### Disturbance and management history

The Liangshui National Nature Reserve experienced a certain degree of human-mediated disturbance before its establishment. During the early stage of forest development, the area was managed by the Dailing Forestry Experimental Bureau, and timber harvesting began in 1953, mainly in the form of clear-cutting. In March 1958, Northeast Forestry College, now Northeast Forestry University, established the Liangshui Experimental Forest Farm in this area as a teaching, research, and field-practice base. Since then, large-scale logging has largely ceased, and only limited experimental final cutting and tending cutting have been conducted in some areas for teaching and scientific research purposes. After Liangshui was approved as a nature reserve by the former Ministry of Forestry in 1979, forest management activities were further restricted, with only small amounts of tending cutting allowed in specific areas (Zhang, 2007).

The study plots were located in compartment 19 of the reserve, which represents one of the relatively well-preserved old-growth broad-leaved Korean pine forest areas. According to available local management information, this area experienced patch clear-cutting and light selective cutting around 1956 (Li, 1988), but the cutting intensity was low and no further logging activities occurred there after. During field surveys, no obvious evidence of historical logging, such as stumps, skid trails, or artificial regeneration, was observed within the study plots. Therefore, the selected plots were regarded as stands with weak recent human disturbance, where current stand structure and regeneration patterns are mainly shaped by natural regeneration and succession processes. Nevertheless, low-intensity human activities, such as long-term teaching and research activities and the collection of non-timber forest products, including mushrooms and ferns, may occur in the reserve, but these activities did not appear to substantially alter the stand structure of the study plots.

### Plot establishment and survey

Four standard plots (each 2,500 m^2^ in size, 50 m × 50 m) were established within the broad-leaved Korean pine forest in the core area of the Liangshui Nature Reserve. To reduce potential spatial dependence among plots, the distance between any two standard plots exceeded 500 m. The plots were located on middle to lower slope positions and laid out along contour-parallel directions. The measurement closure error was strictly controlled within 1/200 (Fan et al., 2013; Du et al., 2025c). The plots were situated at elevations ranging from 340 to 400 m, with slopes between 11° and 15°, and the stand volume per unit area ranged from 261 to 344 m^3^/ha. The dominant species, *Pinus koraiensis*, accounted for 50–70% of the stand volume, with *Abies nephrolepis, Picea koraiensis*, *Tilia amurensis*, and *Fraxinus mandschurica* serving as co-dominant species. A contiguous grid sampling method was used (Dong et al., 2020), where each plot was divided into 100 sampling units of 25 m^2^ (5 m × 5 m) each. All trees with a diameter at breast height (DBH) ≥5 cm were individually measured. DBH was recorded using a diameter tape, and tree height was measured with a Vertex IV hypsometer (accuracy: 0.1 m). The relative position of each tree within the sampling unit was recorded to establish a spatial coordinate system. Meanwhile, we focused our investigation on the regeneration of potential canopy tree species (Yang, Lu & Cao, 2014). Here, potential canopy tree species were defined as species with a maximum diameter at breast height greater than 15 cm. According to the Chinese forestry industry standard (LY 1572–2000), regenerating individuals 0.1–1.5 m tall were classified as seedlings (Dong et al., 2020), while taller individuals that had not yet reached the DBH threshold were defined as saplings (Pak et al., 2023). For each seedling and sapling, the species name, height, and spatial position were recorded to provide data support for subsequent analyses of community structure.

### Functional trait measurements

We collected leaf samples for functional trait analysis from seedlings and saplings of each species along transects established around the edges of the study plots. The transects were laid out as follows: at the 1/4 and 3/4 positions along the right side of each of the four plots, a 50 m measuring tape was extended outward from the plot boundary in a direction parallel to the contour lines to serve as the centerline of the transect. From this centerline, the transect was extended 5 m both uphill and downhill to form a belt-shaped sampling area (See Fig. S2). Eight transects were established across the four plots. Sampling seedlings and saplings along these transects reduced the randomness of individual selection. The eight transects generally ensured that at least 10 individuals of each tree species were sampled. For species with fewer than 10 sapling individuals within the transects (*e.g*., *Fraxinus mandshurica* and *Ulmus laciniata*), additional individuals were selected from areas adjacent to the transects. Leaf samples for trait analysis were collected according to the guidelines in the study by Pérez-Harguindeguy et al. (2016), selecting intact, sun-exposed leaves free from disease or insect damage. However, in this study, we did not exclude individuals growing under the forest canopy. Instead, sampling was conducted progressively along each transect from the starting point to the end, with individuals sampled in the order of their appearance. Three to five leaf samples were collected for each selected individual. Data regarding leaf traits of regenerating seedlings and saplings for each tree species were collected during the peak leaf period (July 15 to August 1). Leaf thickness was measured and recorded in the field. To prevent moisture loss from affecting trait measurements, the leaves were quickly placed in pre-weighed, screw-cap centrifuge tubes and labeled with individual IDs. Fresh weight was measured on the same day using the subtraction method. After weighing, the centrifuge tubes were opened, and the leaves were scanned using a BenQ-5560C color scanner to enable later extraction of leaf area, length, and width using Photoshop CS 8.01 (Adobe Systems Inc., San Jose, California, USA). After scanning, the leaves were returned to the centrifuge tubes and oven-dried to a constant weight. After drying and weighing, the leaf samples from each tree species were pooled, ground, and sieved for chemical trait analysis. Total carbon and nitrogen contents were measured using a carbon-nitrogen analyzer (vario Macro cube, Elementar, Germany), while the phosphorus and potassium contents were determined using inductively coupled plasma emission spectrometry (ICPS-7500; Shimadzu, Japan). Four parallel measurements were performed on composite samples from the seedling and sapling stages of each species to ensure that the results were reliable. The analytical principles and detailed procedures of the instruments used for leaf chemical trait measurements are provided in Appendix 1. In total, 15 leaf traits were measured: leaf length (LL), leaf width (LW), leaf area (LA), specific leaf area (SLA), leaf dry matter content (LDMC), carbon content per unit mass (C_mass), nitrogen content per unit mass (N_mass), phosphorus content per unit mass (P_mass), potassium content per unit mass (K_mass), nitrogen content per unit area (N_area), phosphorus content per unit area (P_area), carbon-to-nitrogen ratio (C/N), carbon-to-phosphorus ratio (C/P), and nitrogen-to-phosphorus ratio (N/P). The sample sizes and leaf trait characteristics of regenerated seedlings and saplings for each tree species are provided in Appendix Table S1. All field surveys, including plot establishment, regeneration census, and functional trait sampling, were conducted with permission from the Liangshui National Nature Reserve, under field permit approval number LS20190501.

### Data analyses

#### Analysis of aggregation scale and size in local regeneration communities

Local regeneration communities were defined as spatially aggregated fragments of the regional regeneration community (Wassel & Myers, 2025), and were hypothesized to exhibit a nested aggregation pattern. To determine the aggregation scale and aggregation size of these local communities, the spatial point patterns of seedlings and saplings were analyzed using Ripley’s *g*(*r*) function, and the observed *g*(*r*) function was then fitted with a homogeneous Thomas process with two nested scales. This analytical framework follows Wiegand et al. (2007), Wiegand, Gunatilleke & Gunatilleke (2007). Detailed procedures are provided in Appendix 2.

#### Functional structure analysis of local regeneration communities

After the small- and large-scale aggregation ranges were determined using a Thomas process with two nested scales, local regeneration communities were delineated between these two aggregation scales by referring to the spatial clustering approach of Plotkin, Chave & Ashton (2002). Specifically, hierarchical clustering with complete linkage was performed based on the spatial coordinates of naturally regenerated seedlings and saplings, and the clustering results were cut at different spatial thresholds to obtain local regeneration communities at different aggregation scales (Podlaski, 2019). To reduce edge effects, local communities located near plot boundaries were excluded. To distinguish community assembly signals at different structural levels, standardized effect sizes of mean pairwise trait distance (*SES.MPD*) were calculated for local regeneration communities using both species abundance data and presence-absence data, representing trait clustering or divergence associated with dominance structure and species composition, respectively (Ding et al., 2021; Yaxley, Skeels & Foley, 2023). In addition, standardized Pielou’s evenness (*SES.E*) was calculated to characterize the degree to which a local community was dominated by one or a few species. All standardized effect sizes were classified into three categories using −1.96 and 1.96 as significance thresholds, and the distribution tendency index D was further used to quantify the tendency of local regeneration communities to fall into different categories across spatial scales (Du et al., 2024, 2025a, 2025b). These calculations were integrated into the R package *M.COMMUNITIES*, and detailed formulas and procedures are provided in Appendix 3.

#### Analysis of the relationship between local regeneration community-weighted mean trait values and the canopy environment

After local regeneration communities were identified with *M.COMMUNITIES*, community-weighted mean leaf area (CWMLAs) were calculated for each functional trait. Convex hulls with a 2.792 m buffer were constructed for each community following Liu et al. (2013), and canopy variables were extracted using *Broadleaf.Korean.pine.LAI*. redundancy analysis (RDA) was used to assess relationships between canopy variables and CWM traits at representative spatial scales. Detailed procedures are provided in Appendix 4.

## Results

### Aggregation scale and size of local regeneration communities

Based on the regeneration survey data from the four plots, and the parameters of the Thomas process with two nested scales were fitted. Figure 2 shows the results. Under the replicated sampling design, the Thomas process with two nested scales provided a good fit to Ripley’s *g*(*r*) functions for both regenerating seedlings and saplings. The coefficients of determination (*R*^2^) for the fitted distribution patterns reached 0.969 and 0.946, respectively. Based on the fitted model parameters, the small-scale aggregation radius for regenerating seedlings was 0.914 m, and the large-scale aggregation radius was 13.630 m. Meanwhile, the small-scale aggregation radius for regeneration saplings was 0.965 m, and the large-scale aggregation radius was 19.126 m.

**Figure 2:**
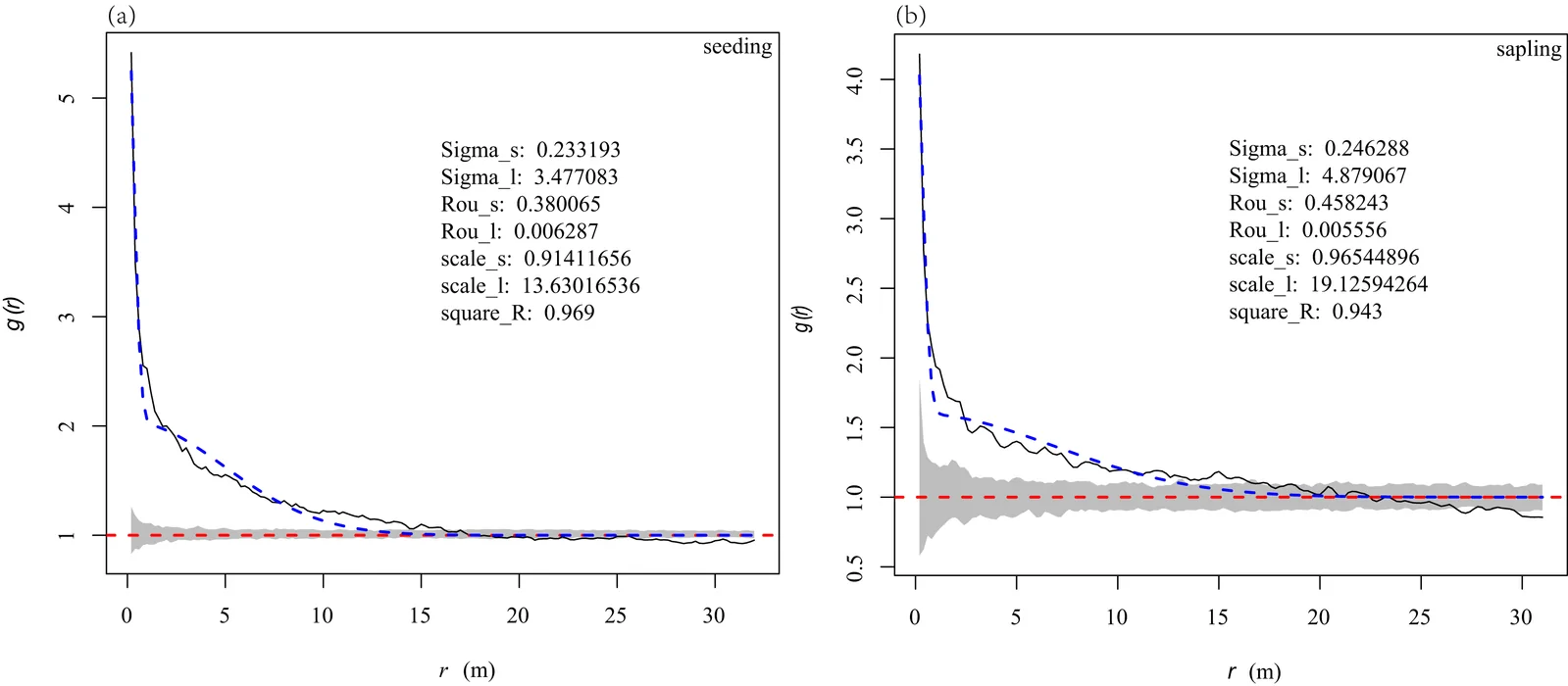
Fitting results of Thomas process with two nested scales for local regenerating seedling and sapling communities.

Considering all four plots together, the mean densities of regenerating seedlings and saplings were 4,641 ± 2,288 individuals.ha^−1^ and 1,298 ± 382 individuals.ha^−1^, respectively. Based on the average densities of seedlings and saplings and their spatial distribution patterns under the replicated sampling design, the average number of individuals per small-scale aggregation patch (including patches with zero individuals) was 1.221 for seedlings and 0.283 for saplings. Using the corresponding aggregation parameters, the average number of small-scale aggregation patches (including empty patches (*i.e*., fitted patches with zero regenerating individuals generated by the Poisson component of the Thomas process)) contained within each large-scale aggregation was 60.453 for seedlings and 82.477 for saplings. Furthermore, the average numbers of non-empty small-scale patches within each large-scale aggregation were estimated as 42.623 for seedlings and 20.329 for saplings. The probability densities of the number of individuals per small-scale aggregation patch and the probability density of the number of small-scale patches within each large-scale aggregation were calculated by substituting the above averages into a Poisson distribution. Figure 3 shows the results.

**Figure 3:**
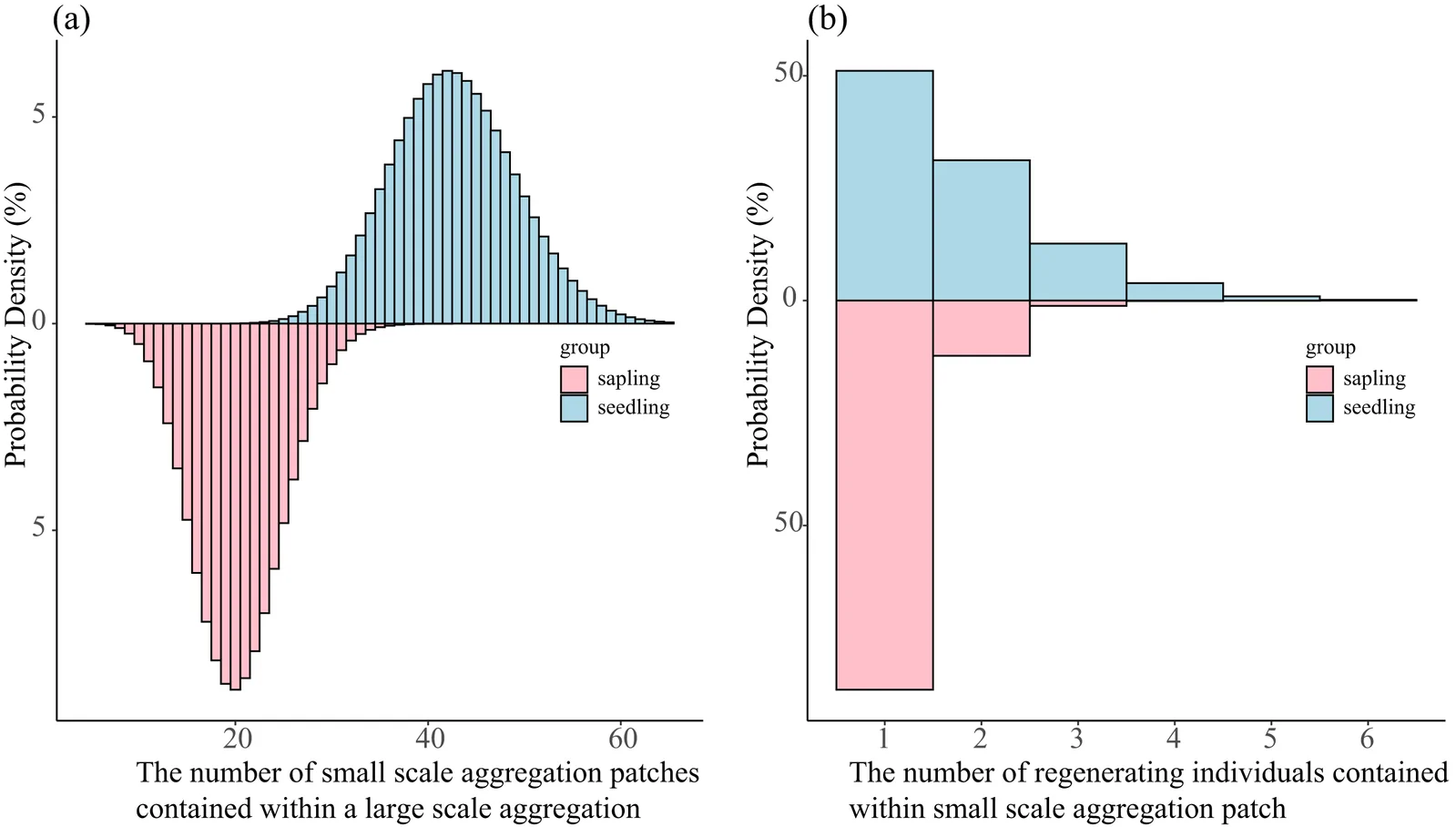
Aggregation intensity of local regenerating seedling and sapling communities at Dual aggregation scales based on Thomas process with two nested scales. (A) The number of small aggregation patches in local communities at the large aggregation scale; (B) the number of regenerating individuals in patches at the small aggregation scale.

As shown in the figure, under the replicated sampling pattern based on four plots, the number of small-scale aggregation patches within the large-scale aggregation patches of local sapling communities was approximately half of that within the large-scale local seedling communities. Similarly, the number of individuals within the small-scale patches was fewer for saplings than for seedlings. After excluding empty patches, the average number of saplings per small-scale aggregation patch was 1.149, while the average number of seedlings was 1.732. This indicates that the size of sapling communities is lower than that of seedling communities at both large and small spatial aggregation scales. This reflects the natural thinning of species in the community during the transition from seedling to sapling stages.

### Functional trait structure of local regeneration communities

Figure 4 presents boxplot-violin plots of standardized metrics for local regeneration communities of seedlings (a) and saplings (b) at different aggregation scales in the broad-leaved Korean pine forest. The metrics included the abundance-weighted and presence-based *SES.MPD* and the *SES.E* within local regeneration communities.

**Figure 4:**
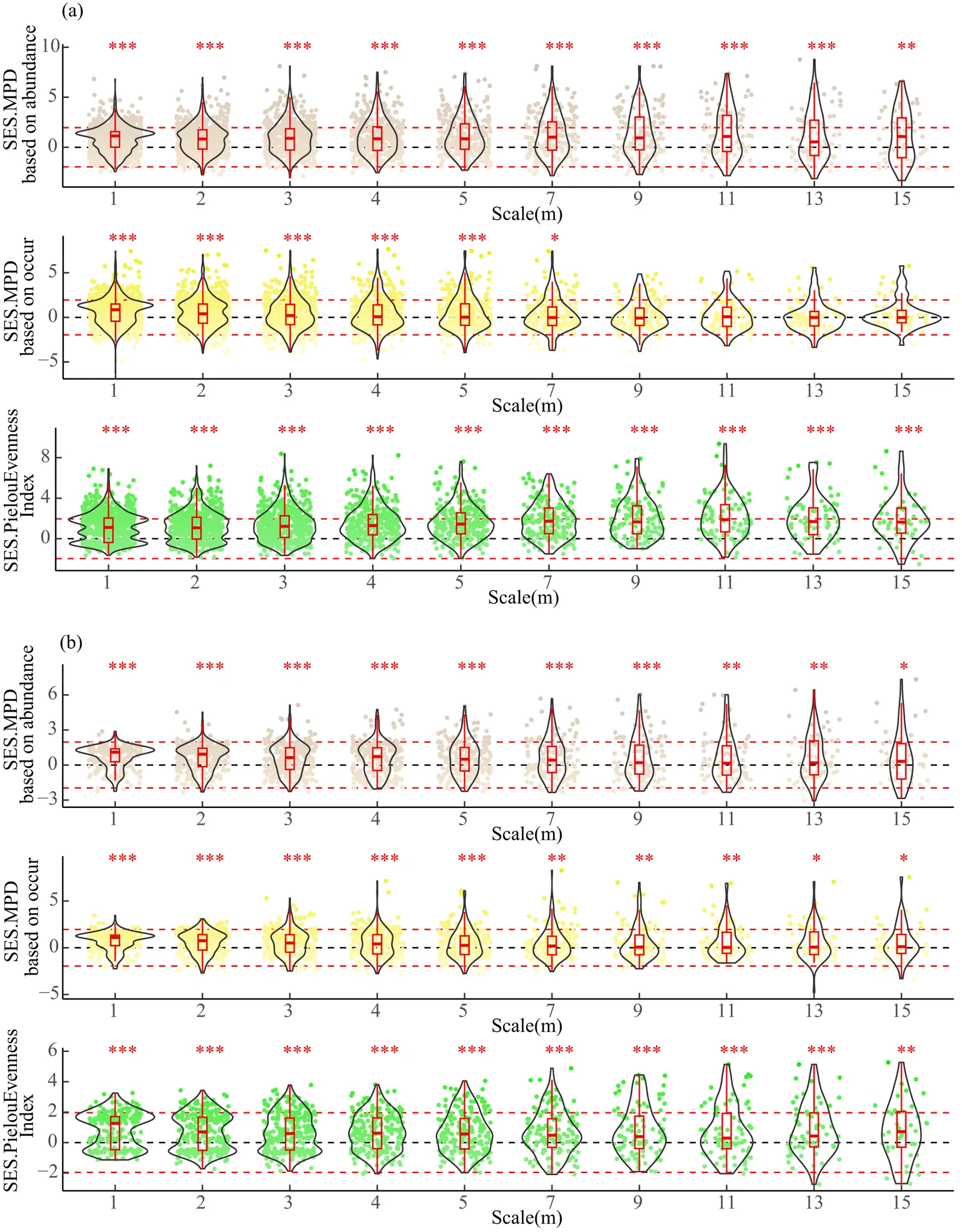
Boxplot-Violin plot of, and of local seedling and sapling communities. (A) Local seedling communities; (B) local sapling communities. ***p < 0.001; **p < 0.01; *p < 0.05; · *p* < 0.1.

In local seedling communities at scales ranging from 1 to 15 m, the abundance-weighted *SES.MPD* was significantly greater than zero (Fig. 4A). Specifically, significance reached the 0.1% level at scales of 1–14 m and the 1% level at 15 m, indicating that leaf trait clustering is generally present in local seedling communities at all scales. For the presence-based *SES.MPD*, the means were significantly greater than zero at scales of 1–7 m, with significance at the 0.1% level from 1 to 5 m and at the 5% level at 7 m. This pattern suggests that the significance level decreases as the aggregation scale of local communities increases, implying that environmental filtering exerts stronger selective pressure on seedling regeneration at smaller spatial scales. This leads to coexistence among seedlings with similar leaf traits. Additionally, the mean *SES.E* for local seedling communities at all scales from 1 to 15 m was significantly greater than zero (*p* < 0.001). Combining the results from the presence-based *SES.MPD* and the *SES.E* suggests that leaf trait clustering in small-scale local seedling communities is jointly driven by interspecific trait convergence and low species evenness within communities. In contrast, trait clustering in large-scale communities is primarily attributed to low species evenness.

In local sapling communities at aggregation scales from 1 to 15 m, the abundance-weighted *SES.MPD* values were all significantly greater than zero (Fig. 4B). Specifically, significance levels reached 0.1% at scales from 1 to 9 m, 1% at 11 and 13 m, and 5% at 15 m. The presence-based mean pairwise trait distances were significantly greater than zero across all aggregation scales. The significance level was 0.1% at scales from 1 to 5 m, 1% at scales from 7 to 9 m, and 5% at 13 and 15 m. Similar to the local seedling communities, the *SES.E* for sapling communities was significantly greater than zero at all scales (*p* < 0.01). A comparison of the functional trait structures between seedling and sapling communities revealed that the main difference was in the presence-based mean pairwise trait distances at larger aggregation scales. Specifically, sapling communities demonstrated more pronounced clustering in leaf traits among species pairs at larger spatial scales than local seedling communities.

We calculated the distribution tendency for each metric based on the abundance-weighted and presence-based *SES.MPD* as well as the *SES.E* of local seedling and sapling communities (Table 1). The results showed that across all aggregation scales, the distribution tendency degrees of all metrics in both seedling and sapling communities were slightly greater than 1. This indicated that leaf traits among randomly sampled individuals or species pairs exhibited a tendency toward clustering and that species composition in local communities tended toward single-species dominance. At all investigated scales, the distribution tendency degree values of the abundance-weighted *SES.MPD* and the *SES.E* were higher in seedling communities than in sapling communities at the corresponding aggregation scales. Moreover, the distribution tendency degree of the presence-based *SES.MPD* was higher in seedling communities than in sapling communities at smaller spatial scales. This pattern reversed as the aggregation scale increased.

**Table 1:** Distribution tendency degree of SES.MPD_abun,_ SES.MPD_occur_ of leaf traits and SES.E in local seedling and sapling communities at different aggregation scales.

Developmental stage	Distribution tendency degree	1 m	2 m	3 m	4 m	5 m	7 m	9 m	11 m	13 m	15 m
Seedling	D of SES.MPD_abun_	1.073	1.096	1.111	1.126	1.114	1.164	1.176	1.182	1.159	1.153
	D of SES.MPD_occur_	1.057	1.065	1.055	1.066	1.062	1.054	1.040	1.041	1.038	1.071
	D of SES.E	1.150	1.138	1.162	1.173	1.181	1.220	1.228	1.241	1.235	1.204
Sapling	D of SES.MPD_abun_	1.018	1.03	1.041	1.051	1.062	1.074	1.101	1.089	1.123	1.079
	D of SES.MPD_occur_	1.018	1.035	1.047	1.054	1.059	1.059	1.085	1.103	1.114	1.079
	D of SES.E	1.071	1.090	1.086	1.079	1.089	1.086	1.117	1.116	1.140	1.079

### Associations between the canopy environment and local regeneration CWM traits

In the redundancy analysis of local seedling regeneration communities across aggregation scales from 1 to 15 m (Fig. 5), the global coefficient of determination (*R*^2^) describing the relationship between CWM traits and canopy environmental characteristics of regeneration habitats was 0.021, 0.047, 0.104, and 0.206, respectively. These results indicate that canopy environmental variation explained only a small portion of the functional trait variation in local seedling communities, suggesting a strong component of random variation in CWM traits. Figure 5 shows that the first two RDA axes accounted for most of the constrained variation in local seedling communities, indicating that the main structure of the canopy environment–CWM trait relationship was well represented in the two-dimensional ordination space. At the same time, as the aggregation scale increased, the randomness of CWM trait variation decreased, and the influence of deterministic processes such as environmental filtering became more pronounced. Across different aggregation scales, there were consistent relationships between canopy environmental characteristics and CWM traits in local seedling communities. For example, the Local broadleaved LAI was consistently positively correlated with CWM.N_area, CWM.P_area, CWM.C_mass, CWM.C/N, and CWM.C/P, and negatively correlated with CWM.N_mass, CWM.P_mass, and CWM.K_mass. The degree of vertical distribution tendency was positively correlated with local coniferous LAI; both are often positively associated with CWM.LA, CWM.LW, and CWM.SLA, and negatively correlated with CWM.N_area and CWM.P_area. Functional traits within local seedling communities also showed internal patterns of association. For example, CWM.N_mass, CWM.P_mass, and CWM.K_mass, as well as CWM.N/P, were strongly intercorrelated but were negatively correlated with CWM.N_area, CWM.P_area, CWM.C/N, CWM.C/P, and CWM.C_mass. In addition, CWM.LW, LA, and CWM.SLA were strongly positively intercorrelated and negatively correlated with CWM.LT.

**Figure 5:**
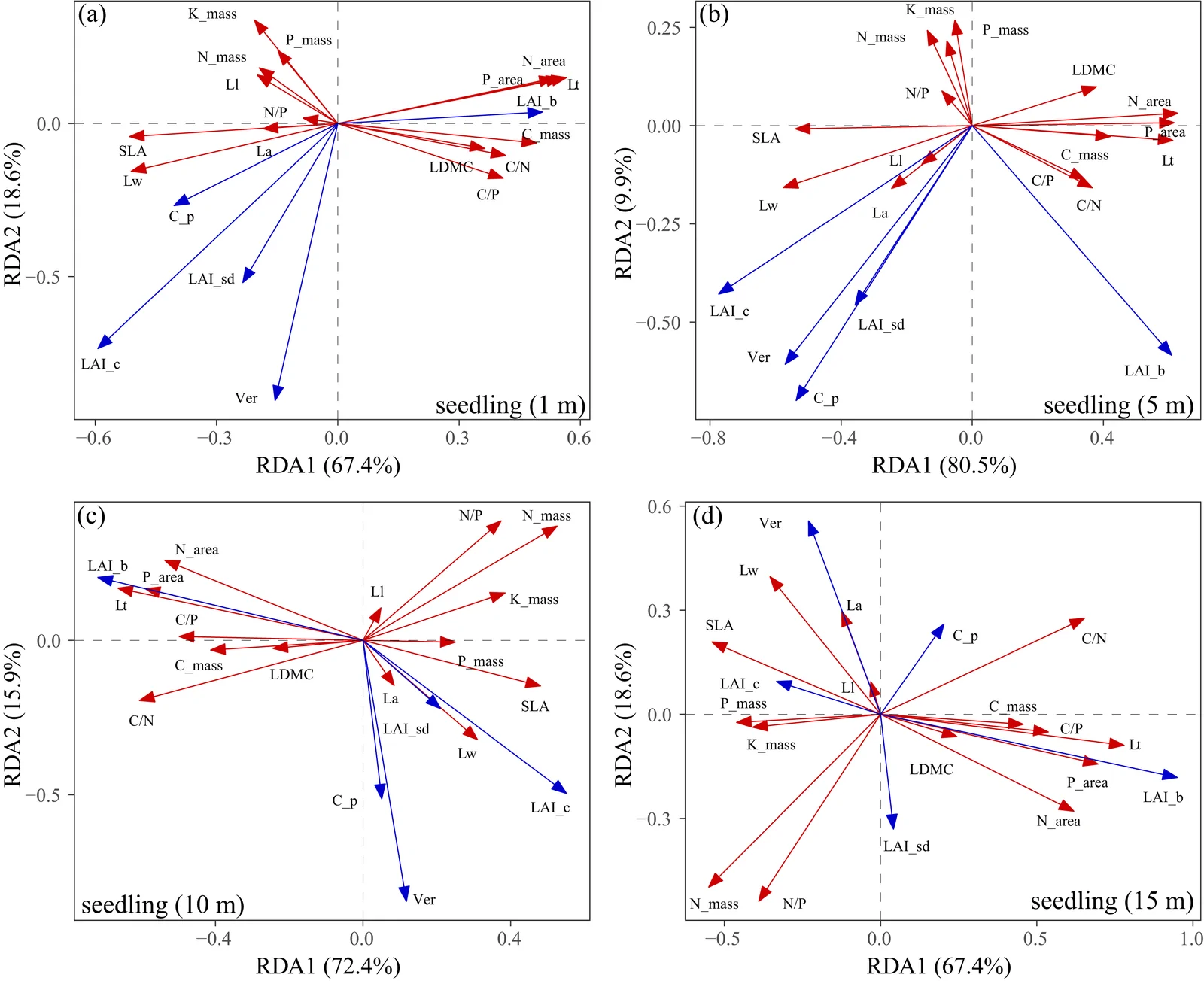
Redundancy analysis of CWM traits and canopy environments in local seedling communities at different aggregation scales. (A), (B), (C), and (D) represent local seedling communities at aggregation scales of 1, 5, 10, and 15 m, respectively. The blue arrow labeled LAI_sd means Local LAI Standard Deviation, C_p means Canopy Percent, LAI_c means Local Coniferous LAI, LAI_b means Local Broadleaved LAI, and Ver means vertical distribution tendency degree, the same below.

In the redundancy analysis of local sapling regeneration communities across aggregation scales from 1 to 15 m (Fig. 6), the global coefficient of determination (*R*^2^) describing the relationship between community-weighted mean traits and canopy environmental characteristics of regeneration habitats was 0.051, 0.123, 0.210, and 0.245, respectively. The first two RDA axes accounted for most of the constrained variation in local sapling communities. Overall, the sapling communities at the same aggregation scales exhibited higher global *R*^2^ values than the seedling communities, indicating that during the transition from seedling to sapling, environmental filtering and species-specific growth performance under different canopy conditions reduce the randomness of trait expression in local communities. Similar to the seedling communities, in the sapling communities at all aggregation scales, the Local broadleaved LAI was positively correlated with CWM.N_area, CWM.P_area, CWM.C_mass, and CWM.C/N. However, compared with the seedling communities, the sapling communities showed a weakly positive or even a negative correlation between CWM.LA, CWM.SLA, and CWM.LW and the degree of vertical distribution tendency as well as the local coniferous LAI. This suggests that broadleaf tree seedlings that become established in areas with lower LAI under the Korean pine canopy have a higher likelihood of growing into saplings.

**Figure 6:**
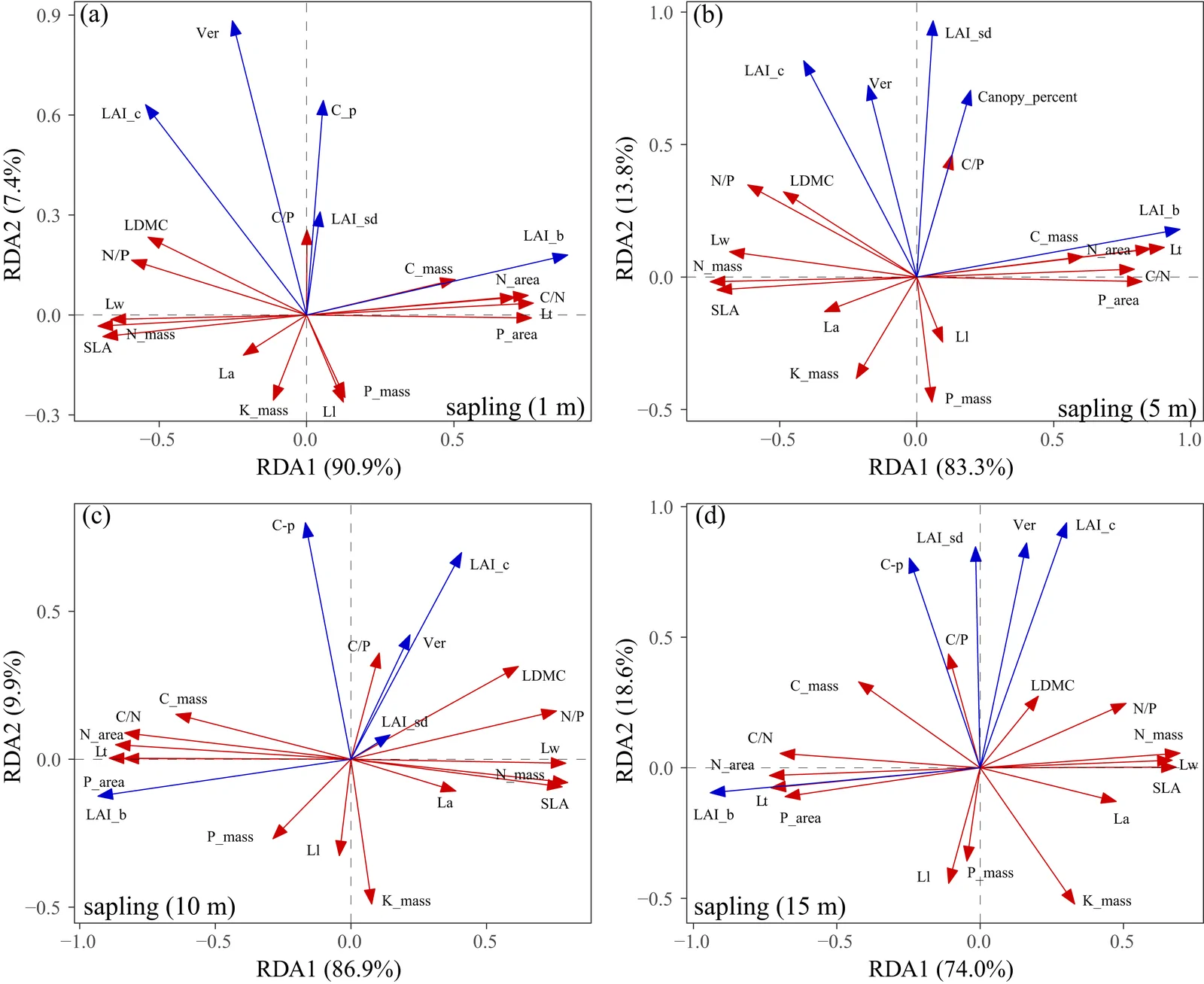
Redundancy analysis of CWM traits and canopy environments in local sapling communities at different aggregation scales. (A), (B), (C), and (D) represent local seedling communities at aggregation scales of 1, 5, 10, and 15 m, respectively. The blue arrow labeled LAI_sd means Local LAI Standard Deviation, C_p means Canopy Percent, LAI_c means Local Coniferous LAI, LAI_b means Local Broadleaved LAI, and Ver means vertical distribution tendency degree, the same below.

## Discussion

### Aggregation scale and size of local regeneration communities

The aggregation scale and size of understory regeneration communities reflect the combined effects of dispersal processes and environmental filtering, and exploring the mechanisms underlying this scale dependence helps to deepen our understanding of natural regeneration processes and community assembly dynamics in climax forests (Asefa et al., 2020). Analyses based on Ripley’s *g(r)* function and Thomas process with two nested scales indicate that local seedling and sapling communities beneath broad-leaved Korean pine forests share a locally nested clustering pattern, with aggregation occurring at approximately 1 and 15 m scales. These results support our first hypothesis that, due to limited seed dispersal and microhabitat heterogeneity, understory regeneration communities exhibit significant spatial aggregation at local scales. Within these forests, the spatial aggregation of regenerated seedlings and saplings at a scale of approximately 1 m may be related to animal seed-caching behavior and the dispersal limitation of propagules. In broad-leaved Korean pine forests, food-hoarding animals such as *Sciurotamias davidianus*, *Tamias sibiricus*, and Nucifraga *caryocatactes* often cache multiple seeds of *Pinus koraiensis* or *Quercus mongolica* at a single storage site (Yu et al., 2013; Li et al., 2019a). Most broad-leaved tree species in Korean pine forests are capable of regenerating *via* sprouting, and this commonly leads to small-scale spatial aggregation patterns that have been observed in many ecosystems (Lu, Zhu & Wang, 2020; Li & Zhang, 2021; Liu et al., 2021). The regenerated seedlings and saplings exhibited spatial aggregation at relatively large spatial scales of approximately less than 15 m. This pattern may be associated with the spatial distribution of the overstory canopy, where aggregation scales are likely influenced by the area formed by multiple overstory trees (Zhan et al., 1984; Wang, Wu & Shi, 1998). In the plots of this study, understory seedlings and saplings generally tended to establish in low-light environments with higher canopy closure and relatively larger LAI, while fewer individuals occurred in the centers of larger canopy gaps (Du et al., 2024, 2025a). This spatial pattern reflects the importance of shade tolerance, a key trait for many tree species in climax forest communities that enables them to establish and maintain their seedling banks under the shaded conditions of the forest canopy (Kursar, 1998; Piovesan & Lüttge, 2018). In broad-leaved Korean pine forests, a competitive network exists among overstory trees, understory regeneration, and shrub–herbaceous layers. The spatial distribution of canopy trees can suppress the growth of understory shrubs and herbs, thereby creating vacant niches that facilitate the establishment of regenerating tree species (Zhang & Wang, 2010). This view is supported by the study by Qiu et al. (2020) who found a significant negative correlation between the abundance of tree seedlings and the herbaceous layer coverage in the Changbai Mountain broad-leaved Korean pine forest. Yao, Zhang & Zhao (2018) analyzed the spatial distribution patterns of dominant tree species in this forest using the *g*(*r*) function and a heterogeneous Poisson model and reported that most species exhibited aggregated distribution patterns at small scales (1–10 m), whereas at scales above 15 m, population spatial patterns tended to shift toward randomness or uniformity. In broad-leaved Korean pine forests, the spatial aggregation scale at the population level (with populations exhibiting an inverse J-shaped structure dominated by younger individuals) is similar to that of regenerating seedlings and saplings at the community level, which includes major tree species (Yao, Zhang & Zhao, 2018). This suggests that the external environmental factors limiting the distribution of dominant tree species during regeneration may be relatively similar, resulting in a certain degree of homogeneity in the regeneration patterns across species. Under the replicate sampling model, the local sapling communities exhibited lower aggregation intensity than the local seedling communities at both the larger and smaller aggregation scales. This trend of decreasing aggregation intensity with advancing developmental stages has also been observed at the population level in common tree species within Korean pine forests. The underlying reason for this phenomenon may be that tree species tend to produce numerous propagules to preemptively occupy available niches, adopting a survival strategy that increases the likelihood of successful random establishment by elevating the initial density of seedlings. The interspecific differences primarily reflect variation in the strength of this tendency. As seedlings grow, their demand for resources increases, and environmental filtering, along with intraspecific and interspecific competition, leads to natural thinning of individuals within regenerating populations or communities.

### Scale-dependent assembly processes in local regeneration communities

The difference between abundance-weighted functional diversity indices and functional diversity indices based solely on species presence can be used to quantify the strength of dispersal processes in community assembly as expressed through their effects on species abundance structure (De Bello et al., 2016). The intensity of dispersal effects is typically reflected in the abundance patterns of dominant species within the local community; under dispersal limitation, local communities tend to be established and maintained primarily by species that are abundant in nearby source communities, whereas low-abundance or distant species are less likely to colonize, resulting in local species composition and abundance patterns that more strongly mirror the structure of proximate source communities (Mouquet & Loreau, 2003; Canham et al., 2014; Suzuki & Economo, 2021). More generally, differences between abundance-weighted and presence-based MPD capture variation in the relative abundance structure of species within local communities (Xu et al., 2016), thereby providing a functional-trait-based proxy for dispersal-driven mass effects in community assembly. These abundance-structure effects can be further summarized using classical abundance-based indices, such as species evenness or the Simpson diversity index, which reflect the degree to which community composition is shaped by dominant *vs*. rare species (Heip, Herman & Soetaert, 1998). Within this framework, we evaluated how dispersal limitation and environmental filtering jointly shape trait structure across aggregation scales. In this research, the abundance-weighted *SES.MPD* and the *SES.E* values of local seedling communities were significantly greater than zero across all aggregation scales; in contrast, the presence-based *SES.MPD* was significantly greater than zero only at smaller aggregation scales. This indicates that at smaller spatial scales, local microenvironmental conditions are relatively homogeneous, and environmental filtering plays a dominant role in driving the convergence of leaf traits within seedling communities. As spatial scale increases, environmental differences among microhabitats are progressively integrated, leading to a weakening of trait convergence signals among randomly sampled individuals. Under these conditions, the trait structure of local seedling communities increasingly reflects the effects of propagule dispersal limitation. Previous studies have shown that, across forest ecosystems from cold-temperate to tropical regions, the seeds of most tree species are primarily distributed within approximately 20 m of the parent tree (Norghauer & Newbery, 2015; Soliani et al., 2025), whereas the spatial range of variation in canopy leaf area index (LAI) in broad-leaved Korean pine forests is about 5.108 m (Du et al., 2025b). This mismatch between dispersal scale and canopy structural scale can, from an indirect perspective, support a pattern in which trait convergence is mediated by environmental filtering at smaller spatial scales and by dispersal limitation at larger spatial scales. Similar to local seedling communities, the values of both the abundance-weighted *SES.MPD* and the *SES.E* were significantly greater than zero across all aggregation scales in local sapling communities. However, unlike in seedling communities, the presence-based *SES.MPD* in sapling communities was also significantly greater than zero at larger aggregation scales. This suggests that during the transition from seedlings to saplings, the effects of environmental filtering accumulate over time, leading to a tendency for species with similar leaf traits to occur together in local sapling communities. This pattern further suggests that constraints imposed by environmental filtering accumulate across ontogenetic stages and may operate as a general mechanism shaping community assembly (Kunstler et al., 2012; Li et al., 2019b). The degree of distribution tendency for the abundance-weighted *SES.MPD* and the *SES.E* was higher in local seedling communities than in sapling communities at the same aggregation scale. A possible explanation for this phenomenon is that as seedlings grow into saplings, their demands for resources and space increase, thereby intensifying intraspecific competition. This explanation is supported by the study by Yao et al. (2016), which indicated that negative density dependence was widespread among woody plant populations in old-growth Korean pine forests. At smaller aggregation scales, the degree of distribution tendency of the presence-based *SES.MPD* in local sapling communities decreased, reflecting not only intraspecific competition but also competitive exclusion among species with similar survival strategies. In contrast, the increase in this tendency at larger aggregation scales may be largely attributed to environmental filtering dominated by the canopy structure. Taken together, these results support our hypothesis that community structure is jointly shaped by dispersal limitation and environmental filtering, while limiting similarity further constrains species coexistence, and that the relative importance of these processes varies across aggregation scales. These findings demonstrate that different ecological mechanisms mediate the assembly of understory local regeneration communities at different spatial scales (Wright, 2002).

### Associations between canopy environmental heterogeneity and regeneration community composition

Canopy conditions can shape trait syndromes of regenerating individuals by altering understory light availability, temperature, and moisture at local scales. In addition, canopy composition partly reflects the identity and spatial distribution of local parent trees, and thus is indirectly linked to propagule dispersal effects as well as the competitive pressure exerted by overstory trees on nearby regenerating individuals. These influences can be regarded as deterministic components of community assembly; meanwhile, stochastic processes may also play an important role during the assembly of local regeneration communities. To gain mechanistic insights into community assembly, we applied redundancy analysis (RDA) to test whether canopy environmental gradients are associated with shifts in trait composition, quantified as CWM leaf traits of seedling and sapling assemblages across aggregation scales. The *R*^2^ values from the RDA of the CWM trait values of local seedling and sapling communities were generally low, ranging from 0.021 to 0.245. Because RDA quantifies the proportion of trait variation explained by the measured canopy environmental variables, these relatively low R^2^ values suggest that canopy-related environmental filtering represented a weak deterministic component of local regeneration community assembly. This indicates that, during the assembly of local regeneration communities beneath broad-leaved Korean pine forests, variation in functional traits is likely to arise largely from stochastic effects that are difficult to measure directly, with neutral processes occupying an important role in community assembly, while the explanatory power of canopy environmental conditions for trait structure is relatively limited. Previous studies have observed such community assembly rules being dominated by stochastic processes in the overstory stand of the broad-leaved Korean pine forests (Tan et al., 2017), as well as in the regeneration layers of other forest ecosystems (Shibata et al., 2010). With increasing aggregation scales, the *R*^2^ values from the RDA of the CWM trait values of local seedling and sapling communities showed an increasing trend; furthermore, the values were higher for local sapling communities than for seedling communities. This increase in explanatory power with spatial aggregation scale may be because local communities at larger aggregation scales contain nested smaller-scale communities, and these smaller-scale communities are more strongly influenced by neutral ecological processes, such as dispersal limitation and priority effects. As a result, the dominant species within these communities exhibited a greater degree of randomness. In contrast, in larger-scale local communities, the stochastic effects nested within small-scale communities tend to cancel out, whereas deterministic effects such as environmental filtering are retained. Tan et al. (2017) noted that the assembly of local communities in temperate forests of northeastern China was jointly driven by niche-based processes dominated by environmental filtering and neutral processes dominated by dispersal limitation, with the latter playing a leading role. Moreover, the smaller the spatial scale of the local community, the stronger the influence of dispersal limitation, whereas the effect of environmental filtering becomes more prominent at larger spatial scales, consistent with our results. At the same spatial aggregation scale, the higher *R*^2^ values for CWM leaf traits for local sapling communities than seedling communities may be attributed to the fact that, relative to seedlings, saplings are exposed to canopy environmental filtering and have had a longer period to respond to variation in canopy conditions. The cumulative effect of long-term canopy influence plays a more prominent role in the community assembly process of saplings. Spasojevic et al. (2014), in their comparative study of temperate forests in the Ozarks of Missouri, USA, found that the CWM trait values of mature trees exhibited a stronger response to soil environmental variables than those of saplings. At the same time, the functional dispersion index of traits was higher in sapling communities than in mature tree communities, indicating that stochastic processes played a greater role in the assembly of sapling communities. These findings were consistent with our results, demonstrating that the influence of environmental filtering increases as plants in the community mature. Overall, from the RDA results, canopy-related environmental filtering was detectable but relatively weak in explaining overall trait variation.

In local seedling and sapling communities across different aggregation scales, the relationships between various canopy environmental factors and CWM leaf trait values exhibited similar response patterns. For example, the local broadleaved LAI in the habitats where these communities were established was consistently positively correlated with CWM.P_area, CWM.N_area, CWM.C_mass, CWM.C/N, and CWM.LT. In contrast, the local broadleaved LAI was negatively correlated with CWM.N/P, CWM.N_mass, CWM.LW, CWM.LA, and CWM.SLA. Additionally, the local broadleaved LAI tended to be negatively correlated with the local coniferous LAI. These patterns are likely related to the tendency of broadleaf species to regenerate under coniferous canopy patches and coniferous species to regenerate under broadleaf canopy patches (Pak et al., 2023; Du et al., 2025a). This reflects a temporal niche differentiation in photosynthetic accumulation between the upper canopy and the understory regeneration layer (Zhou, 2019), as well as the operation of the principle of limiting similarity. At the same time, these correlations also changed with ontogeny, indicating ontogeny-dependent responses. Compared with seedling communities, the positive correlations of CWM.LA, CWM.SLA, and CWM.LW with the degree of vertical distribution tendency of the canopy and the local coniferous LAI tended to weaken or even shift to negative correlations in sapling communities. In addition, CWM.P_mass and CWM.K_mass increased markedly in habitats where both local coniferous and broad-leaved LAI were low. This may be attributed to the fact that in relatively open understory environments, broadleaf species regenerate and grow more rapidly than coniferous species, resulting in sapling communities characterized by R-strategy traits, *i.e*., short lifespan and high growth rates (Wright et al., 2004; Reich, 2014). This pattern is also consistent with the gap-phase dynamics of broad-leaved Korean pine forests, where broadleaf species rapidly grow following gap formation and subsequently provide shade for the regeneration of coniferous species.

### Ecological implications for forest dynamics and management

Analyses of understory regeneration mechanisms in broad-leaved Korean pine forests have revealed several ecological patterns. However, how these patterns can be translated into effective management strategies for primary forests, secondary forests, and planted forests within the same region remains a topic worthy of further investigation. For secondary forests, management objectives are typically focused on accelerating successional progression and shortening the time required for recovery to a climax community in terms of both structure and function. However, the scarcity of regeneration sources for climax tree species in the understory is widely recognized as one of the key factors constraining successional progression in secondary forests (Ratanapongsai, 2020; Joyce, Zahawi & Holl, 2025). Based on this understanding, previous studies have proposed the artificial sowing or enrichment planting of climax tree species beneath the canopy of secondary forests to compensate for the limited availability of propagules caused by restricted natural dispersal, thereby facilitating the establishment and regeneration of climax species (Li et al., 1993; Cong et al., 2011). This management practice is considered capable of significantly increasing forest growing stock per unit area, enhancing biodiversity, and increasing the structural complexity of the forest canopy. The results of this study further indicate that coniferous species that become dominant in the late successional stages tend to establish preferentially beneath canopy patches formed by broadleaved tree species (Pak et al., 2023; Du et al., 2025a), whereas the community-forming species in secondary forests are mostly broadleaved trees with shorter lifespans and faster growth rates. This management approach of underplanting coniferous species beneath broadleaved canopies closely resembles the regeneration patterns observed during gap dynamics in primary forests. Specifically, following gap formation, broadleaved species often colonize vacant niches first and, over a certain period, provide shading and protective shelter for slower-growing, more shade-tolerant coniferous species (Zang, Liu & Dong, 1999). These findings indicate that this regeneration mechanism not only provides a theoretical basis for the management of secondary forests, but that its underlying principles can also be applied to the transformation of broadleaved plantations into multilayered structures and to close-to-nature forest management practices. For the regeneration of broad-leaved species in the understory of conifer plantations, they should not be simply regarded as “weeds” and removed (Seliger et al., 2023; Du et al., 2024). When overstory coniferous trees reach harvesting or regeneration standards, artificial canopy openings can be created through selective cutting or directional harvesting, thereby simulating the natural process of gap formation in forests (Puettmann et al., 2016; Seliger et al., 2023). By then, the established broad-leaved tree species in the understory are able to quickly take advantage of the improved light conditions for rapid growth, and further, by regulating the understory microenvironment and enhancing site conditions, they create favorable conditions for the subsequent regeneration of conifer species. This process ultimately drives the stand structure from a single layer toward multiple layers, achieving sustainable forest management (Zang, Guo & Gao, 1998; Zang, Liu & Dong, 1999). This study further indicates that understory regenerating individuals exhibit an aggregation range of approximately 15 m at larger spatial scales, a scale that corresponds closely to the spatial aggregation scale of LAI in broad-leaved Korean pine forests (Du et al., 2025b). This result suggests that, during artificial gap creation or selective logging operations, the formation of excessively large and continuous canopy gaps at a local scale should be avoided, as such gaps may induce abrupt changes in the understory microenvironment and thereby hinder the regeneration and establishment of target tree species. Therefore, in practical forest management, regulating the intensity and spatial configuration of selective harvesting can reduce the concentrated removal of adjacent trees, thereby allowing canopy gap sizes to more closely approximate the spatial scales associated with natural regeneration processes. Based on the dispersal patterns of seed rain and vegetative sprouting, it is generally accepted that the dispersal probability of propagules decreases with increasing distance from the parent tree (Nathan & Muller-Landau, 2000; Wang et al., 2019). However, our results show that, for both coniferous and broad-leaved species, regenerating individuals exhibit a certain degree of spatial repulsion from overstory trees belonging to closely related taxa. This pattern is consistent with findings from previous studies conducted in broad-leaved Korean pine forests (Pak et al., 2023; Wang & Guo, 2019). This phenomenon suggests that, under the canopy of overstory trees, regenerating individuals of closely related species may experience higher mortality risks, thereby constraining their successful establishment. In practical forest management, targeted transplantation of regenerating individuals from unfavorable local habitats to microhabitats that better meet their ecological requirements can be implemented, thereby improving survival rates, reducing the need for repeated replanting, and potentially accelerating forest stand successional development (Reis et al., 2024; Prieto-Rodao, Ricker & Martínez-Ramos, 2023). In addition, clear limiting similarity effects were detected in local sapling communities at aggregation scales below 5 m, indicating that competitive pressure among functionally similar or closely related individuals increases markedly at short spatial distances. This phenomenon has been widely reported across a range of forest ecosystems (Yılmaz et al., 2019; Zhao et al., 2022). From a forest management perspective, this finding suggests that understory planting, seedling transplantation, and tending operations should carefully consider the spatial configuration of individuals. Specifically, high-density arrangements at very fine spatial scales should be avoided to reduce competition arising from close inter-individual proximity, thereby enhancing the growth performance and survival probability of regenerating individuals.

In addition, the application of regeneration patterns in forest management should be considered in relation to stand developmental stage and the frequency of management interventions. Taking human-managed stands as an example, tending and cleaning operations in young stands usually reduce competition from herbs and shrubs against target tree seedlings through moderate shrub cutting and weeding, thereby improving early growth conditions. However, excessively frequent cleaning may reduce the diversity of associated species and non-target regenerating individuals, weakening the potential for the future development of multilayered stand structures (Deng et al., 2020; Zhou et al., 2016). Therefore, during tending operations, a certain proportion of regenerating individuals of associated tree species and potentially valuable non-target regenerating individuals may be retained according to management objectives, while differential retention across tending stages can gradually promote the formation of a more balanced age structure and spatial mosaic pattern (Holmström et al., 2016). In middle-aged and near-mature stands, conventional thinning is mainly intended to reduce competition among trees and provide better growing space for target tree species. However, thinning planning should not focus solely on the growth of retained trees, but may also incorporate the suitability of understory regeneration environments into management design (Yang et al., 2023). Based on studies of regeneration patterns, stand density, canopy openness, and understory light conditions can be regulated to create suitable microenvironments for the natural regeneration of different tree species, thereby optimizing species composition, stand structure, and age gradients. At the mature stand stage, common commercial harvesting methods include clear-cutting and selective cutting. Compared with clear-cutting, selective cutting retains part of the upper canopy trees, which can avoid abrupt changes in the understory environment and help maintain seed-source supply and the continuity of natural regeneration. Based on a more detailed understanding of regeneration mechanisms, selective cutting should not be regarded only as a timber harvesting method, but also as an important approach for regulating canopy gap formation and the development of regeneration patches (Remeš, 2006). Where management conditions allow, repeated shelterwood entries can be used to regulate the size and spatial distribution of canopy gaps according to the light requirements of different regenerating tree species. This can create gradient understory microenvironments and induce the formation of regeneration patches at different developmental stages, allowing newly established regeneration patches, sapling patches, and more advanced regeneration patches to gradually form a spatial mosaic within the stand (Malcolm, Mason & Clarke, 2001). This approach can partly simulate gap-driven patch regeneration in primary forests, promote continuous regeneration of diverse tree species, enrich stand structure, and provide ecological reference for continuous-cover forestry. Future studies should further clarify the optimal matching among gap size, individual spatial arrangement, and natural regeneration outcomes under different stand developmental stages and management intervention frequencies.

## Conclusions

This study analyzed the assembly mechanisms of local seedling and sapling regeneration communities across different aggregation scales under broad-leaved Korean pine forests, thereby addressing the insufficient attention given in previous studies to the assembly mechanisms of small-scale regeneration communities in broad-leaved Korean pine forests. The main findings are as follows. The assembly of local regeneration communities in the Korean pine forest is dominated by stochastic processes. Among deterministic processes, dispersal limitation leads to spatial and trait aggregation of regeneration communities across all spatial scales; canopy-driven environmental filtering accumulates with regeneration development, and limiting similarity plays a more pronounced role in local sapling communities than in seedling communities at smaller spatial scales. Differences in interspecific ontogenetic strategy lead to variation in the trait–canopy relationships between local seedling and sapling communities. These results provide an ecological basis for understanding regeneration patch formation, the maintenance of functional trait structure, and natural regeneration processes in broad-leaved Korean pine forests. They may also provide management references for secondary forests with broad-leaved Korean pine forests as a near-natural management target, as well as for plantations that cultivate the main tree species of broad-leaved Korean pine forests. However, this study has certain limitations. Functional traits were mainly represented at the species level, and intraspecific trait variation along local environmental gradients was not fully incorporated; meanwhile, this study was mainly based on spatial observational data and lacked long-term continuous monitoring of regeneration dynamics. Future studies should integrate individual-level trait measurements, long-term monitoring, and comparative analyses under different management disturbances to further clarify the assembly mechanisms of locally regenerating communities in broad-leaved Korean pine forests and their implications for forest management.

## Appendix

### Appendix 1 Analytical principles and procedures for leaf chemical trait measurements

#### Determination of total carbon and total nitrogen

Total carbon and total nitrogen were determined using a carbon-nitrogen analyzer (vario Macro cube, Elementar, Germany). The instrument determines carbon and nitrogen based on the Dumas combustion method and the adsorption–desorption principle. Briefly, air-dried and uniformly ground plant samples were weighed and wrapped in tin boats. The samples were combusted under oxygen-rich conditions, during which carbon was oxidized to CO_2_ and nitrogen was converted into N_2_ and NO_x_. The mixed gases were carried by helium through a reduction tube containing reduced copper, where NO_x_ was reduced to N_2_. After moisture removal, CO_2_ and N_2_ were separated on a chromatographic column and quantified by the detector. The percentages of total carbon and total nitrogen were calculated after calibration using standard curves.

#### Determination of total phosphorus and total potassium

Total phosphorus and total potassium were determined after acid digestion of plant samples. Air-dried and uniformly ground plant material was digested with concentrated nitric acid and perchloric acid on an electric hot plate until the digestion solution became clear. Blank samples were digested simultaneously to correct for reagent errors. After cooling, the digestion solution was diluted to a fixed volume, and total phosphorus and total potassium contents were measured using inductively coupled plasma optical emission spectrometry. The final contents of total phosphorus and total potassium were calculated based on the dry mass of the plant samples.

### Appendix 2 Aggregation scale and size of local regeneration communities

We hypothesized that local regeneration communities under broad-leaved Korean pine forests would exhibit multiple spatial aggregation scales in a nested structure. The smaller aggregation scales nested within larger scales may result from clumped regeneration caused by scatter-hoarding animals’ seed caching and basal sprouting. In contrast, the formation of larger aggregation scales may be attributed to environmental filtering and reduced competition from canopy-suppressed shrubs and herbs. It should be clarified that, in some fine-scale local units where regenerating individuals are sparse, species composition may be relatively homogeneous, and in some cases only a single species may occur. Therefore, in this study, the term “local regeneration community” is used to denote a spatially aggregated unit of understory regeneration, which essentially represents a local fragment of the regional regeneration community rather than an independent community entity at the population or individual level, and is used to explore the mechanisms by which the regional community is assembled in local space (Wassel & Myers, 2025). Based on this, we adopted the homogeneous Thomas process with two nested scales, following the studies by Wiegand et al. (2007), Wiegand, Gunatilleke & Gunatilleke (2007), to analyze the aggregation scales of local regeneration communities. The analysis involved the following steps.

First, we treated the four plots as replicates and analyzed the overall spatial point patterns of regenerating seedlings and saplings using Ripley’s *g*(*r*) function (Law et al., 2009). Because all four plots were square and had equal side lengths, we calculated the observed *g*(*r*) values from replicate sampling and the upper and lower bounds of the null model envelope under the assumption of complete spatial randomness (CSR) using Eq. (1). The following formula represents the observed value of the replicate sampling *g*(*r*) function or the upper/lower bound of the null model envelope:

(1) $${\hat g}\left( {r} \right)={{\mathop \sum \nolimits_{{ m = 1}}^{ 4} {{ g}_{m}}{(r)}} \over {4}},$$

where *m* denotes the plot number, *g*_*m*_(*r*) is the observed value or the upper/lower bound of Ripley’s *g*(*r*) function for the *m*th plot, and *r* represents the spatial scale.

Wiegand et al. (2007), Wiegand, Gunatilleke & Gunatilleke (2007) found that the homogeneous Thomas process with two nested scales could effectively fit Ripley’s *g*(*r*) function when analyzing the spatial distribution of juvenile individuals of several tropical tree species. The relationship between this process and Ripley’s *g*(*r*) function is described by the following equations:

(2) $${g}\left( {{r},{{\sigma }_{s}},{{\sigma }_{l}},{{\rho }_{s}},{{\rho }_{l}}} \right)={1 }+{{{ h(r,}\sqrt {2} {{\sigma }_{s}})} \over {{{\rho }_{s}}}}+{{{ h(r},\sqrt {{2}{{\sigma }_{s}}^{2}+{2}{{\sigma }_{l}}^{2}} )} \over {{{\rho }_{l}}}},$$

(3) $${h}\left( {{ r,\sigma }} \right)={{{ {\rm exp}( - 0.5}{{r}^{ 2}}/{{\sigma }^{ 2}})} \over {{2\pi }{{\sigma }^{ 2}}}}.$$

In Eqs. (2) and (3), ${g}\left( {{ r},{{ \sigma }_{ s}},{{\sigma }_{ l}},{{\rho }_{s}},{{\rho }_{l}}} \right)$ represents the calculated value of Ripley’s *g*(*r*) function at different spatial scales; ${{ \sigma }_{s}}$, is the shape parameter of the Gaussian distribution at the small aggregation scale, and ${{\sigma }_{ l}}$ is the shape parameter at the large aggregation scale. The parameter ${{ \rho }_{ s}}$ is related to the number of individual plants within small-scale aggregations, and ${{ \rho }_{ l}}$ corresponds to the number of small-scale patches within large-scale aggregations. The method for nonlinear fitting of Ripley’s *g*(*r*) function based on Eqs. (2) and (3) has been integrated into the R package *M.COMMUNITIES*. Equations (2) and (3) indicate that both small- and large-scale spatial aggregations follow isotropic bivariate Gaussian distributions. Therefore, the spatial scale encompassing 95% of individuals within small-scale aggregations or 95% of small-scale patches within large-scale aggregations can be estimated using Eq. (4). This value was defined as the spatial scale of aggregation in our analysis (Linnet, 1987).

(4) $${{ S}_{{cluster}}}={1.96}\times{ \sigma \times 2}.$$

In Eq. (4), ${{S}_{{ cluster}}}$ represents the aggregation scale of either small-scale or large-scale aggregations, and σ is the shape parameter of the Gaussian distribution, denoted as ${{\sigma }_{s}}$ for small-scale aggregations or ${{\sigma }_{l}}$ for large-scale aggregations.

In addition, based on the number of regenerating seedlings and saplings within each plot and the plot area, the parameters ${{\rho }_{s}}$ and ${{\rho }_{l}}$ were used to estimate the average number of individuals per small-scale aggregation patches and the average number of small aggregation patches within each large-scale aggregation. The probability distributions of different numbers of individuals per aggregation patch and different numbers of small aggregation patches per large aggregation can be calculated by substituting these values into a Poisson distribution. For detailed methodology, see the studies by Wiegand et al. (2007), Wiegand, Gunatilleke & Gunatilleke (2007).

Because the Poisson distribution includes the probability of zero events, we excluded the probability of having zero regenerating individuals in a small-scale aggregation patch or zero small-scale aggregation patches in a large-scale aggregation.

(5) $${P}\left( {{ X = k}} \right)={{{{ \mu }^{k}}} \over {{ k!}}} \times {{e}^{{- \mu }}}\quad{ k = 0,1,2, \ldots ,n},$$

(6) $${\hat P}\left( {{ X = k}} \right)={{{ P}\left( {{X = k}} \right)} \over {{1} - { P}\left( {{ X = 0}} \right)}}\quad { k} \ne { 0}.$$

In Eqs. (5) and (6), ${P}\left( {{ X= k}} \right)$ and ${\hat P}\left( {{X= k}} \right)$ represent the uncorrected and corrected Poisson distributions, respectively. X denotes the number of individuals within a small-scale aggregation or the number of small aggregations within a large-scale aggregation; k is the specific count; μ is the Poisson parameter, representing the mean number of individuals per small aggregation or the mean number of small aggregations per large aggregation when zero events are included; and e is the base of the natural logarithm.

### Appendix 3 Functional structure analysis of local regeneration communities

After determining the large and small aggregation scales using the Thomas process with two nested scales, we delineated local regeneration communities by referencing the spatial clustering approach described in the study of Plotkin, Chave & Ashton (2002). The delineation scale of local regeneration communities was set between the small and large aggregation scales. Specifically, based on the spatial coordinates of naturally regenerated seedlings and saplings in the understory, hierarchical clustering was performed using the complete linkage method to group individuals into local regeneration communities corresponding to different spatial aggregation scales (Podlaski, 2019). After clustering, the results were segmented at various spatial thresholds to generate local regeneration communities at multiple aggregation scales. These delineated communities had the following property: within a selected scale, the length of the major axis of a local regeneration community (the maximum distance between any two vertices of the convex polygon formed by the individuals’ spatial coordinates) did not exceed the specified scale. To avoid edge effects, local communities with aggregation scales smaller than 10 m and centroids located within 5 m of the plot boundary were excluded from the analysis. For communities with aggregation scales larger than 10 m, those with centroids closer to the plot edge than half the aggregation scale were also excluded.

To separate assembly signals at complementary structural levels, we calculated the standardized effect size of mean pairwise trait distance (*SES.MPD*) using both presence-absence and abundance-weighted data (Ding et al., 2021; Yaxley, Skeels & Foley, 2023). Presenc-absence *SES.MPD* primarily reflects trait filtering at the membership level (*i.e*., which species can occur and persist within a patch), whereas abundance-weighted *SES.MPD* additionally incorporates dominance structure (*i.e*., which co-occurring species become numerically dominant). The difference between the two metrics therefore captures the additional information carried by relative abundance, which may arise from processes that reshape abundance patterns without necessarily changing species membership, such as dispersal-related mass effects (propagule pressure) and other spatial/historical processes.

(7) $${SES}{.}{MP}{{D}_{{abun}}}= - { 1\times }{{{ MP}{{ D}_{{ obs\_abun}}}-{ mean(MP}{{ D}_{{ rand\_abun}}})} \over {{ sd(MP}{{D}_{{rand\_abun}}})}},$$

(8) $${SES}{.}{MP}{{D}_{{occur}}}= - {\rm 1 \times }{{{ MP}{{ D}_{{ obs\_occur}}}-{ mean(MP}{{ D}_{{ rand\_occur}}})} \over {{ sd(MP}{{D}_{{rand\_occur}}})}}.$$

In Eqs. (7) and (8), ${SES}{.}{MP}{{ D}_{{abun}}}$ and ${SES}{.}{MP}{{ D}_{{ occur}}}$ represent the standardized effect sizes of the mean pairwise functional trait distance based on species abundance and species presence in local regeneration communities, respectively. ${MP}{{ D}_{{ obs\_abun}}}$ and ${MP}{{ D}_{{obs\_occur}}}$ are the observed mean pairwise functional trait distances based on species abundance and species presence in local communities, respectively. ${SES}{.}{MP}{{ D}_{{ abun}}}$ refers to the standardized mean pairwise trait distance calculated from a null model. The null model was constructed by generating random communities through sampling species from the regional community (at the plot scale) based on their relative abundances, using the total abundance of the local community as the sample size. ${SES}{.}{MP}{{ D}_{{ occur}}}$ was calculated similarly, but after sampling based on species abundance in the regional community, the abundance data in the random community were converted to binary (presence–absence) data to calculate the mean pairwise functional trait distance. If the relative abundance or composition of tree species in the local community conforms to completely random sampling from the regional community, ${SES}{.}{MP}{{ D}_{{abun}}}$ and ${SES}{.}{MP}{{ D}_{{ occur}}}$ will have Gaussian distributions with a mean of 0 and a standard deviation of 1, with a corresponding 95% confidence interval of [−1.96, 1.96].

Differences between ${SES}{.}{MP}{{ D}_{{abun}}}$ and ${SES}{.}{MP}{{ D}_{{ occur}}}$ can be amplified when relative abundances are strongly skewed, as expected under dispersal-related mass effects in which nearby sources may supply more propagules to local sink patches. Under such conditions, local regeneration communities may exhibit pronounced dominance by one species or a few species, resulting in trait structure that differs between the two metrics. To capture this dominance pattern independently of $SES{.}MPD$, we calculated a standardized species evenness index for each local regeneration community (Heip, Herman & Soetaert, 1998).

(9) $${SES}{.}{E}= - {1}\times{{{{ E}_{{obs}}}-{mean(}{{E}_{{rand}}})} \over {{ sd(}{{E}_{{rand}}})}}.$$

In Eq. (9), ${SES}{.}{E}$ refers to the standardized effect size of Pielou’s evenness of the local regeneration community; ${{E}_{{obs}}}$ is the observed value of Pielou’s evenness in the local community; and ${{E}_{{rand}}}$ represents the Pielou evenness value of randomly assembled local regeneration communities generated by sampling from the species abundance distribution of the regional community (*i.e*., the plot-scale community) using the same total abundance as in the local regeneration community. When species composition and abundance in the local community follow a pattern of completely random sampling from the regional community, ${SES}{.}{E}$ will have a Gaussian distribution with a mean of 0 and a standard deviation of 1, with a 95% confidence interval of [−1.96, 1.96]. A value of ${SES}{.}{E}$ > 0 indicates a tendency toward dominance by a single species in the local community, and ${SES}{.}{E}$ > 1.96 suggests that this dominance tendency is statistically significant; the converse holds for negative values.

As shown in Eqs. (7), (8), and (9), the *SES.MPD* and *SES.E* within local communities—based on species abundance or presence—use thresholds of −1.96 and 1.96 to determine statistical significance. Accordingly, standardized effect size values less than −1.96 are classified as Category 1, values between −1.96 and 1.96 as Category 2, and values greater than 1.96 as Category 3. The degree of distribution tendency ( D) is used to quantify the number of local regeneration communities falling into each category at different spatial scales. The calculation formula for distribution tendency degree is as follows (Du et al., 2024, 2025a, 2025b):

(10) $${D}={{\mathop \sum \nolimits_{{i = 1}}^{N} {(N }\times {{n}_{i}})} \over {{ (N + 1)/2 }\times \mathop \sum \nolimits_{{ i = 1}}^{N} {{n}_{i}}}}.$$

In Eq. (10), where N is the number of categories into which local communities were classified based on their standardized effect size (here, there were three categories), n is the number of local communities in the n-th category, and D is the distribution tendency index, which ranges from 0 to 2. A value of D > 1 indicates that local community classifications tend to fall into Category 3, while a value of D < 1 suggests a tendency for these classifications to fall into Category 1.

The calculation of ${SES}{.}{MP}{{D}_{{abun}}}$, ${ SES}{.}{MP}{{D}_{{occur}}}$, and ${SES}{.}{E}$ of local regeneration communities was integrated into the R package *M.COMMUNITIES*.

### Appendix 4 Associations between the canopy environment and local regeneration CWM traits

The coordinates of seedlings or saplings in each local regeneration community were extracted using the M.COMMUNITIES package, and the community-weighted means (CWMs) of each trait were calculated. Based on the coordinates of individuals in each local regeneration community, the Broadleaf.Korean.pine.LAI package was used to extract canopy environmental characteristics at each location, including the Average Local LAI, Local LAI Standard Deviation, Canopy Percent, Local Coniferous LAI, Local Broadleaved LAI, and degree of vertical distribution tendency. During the extraction process, a convex polygon (convex hull) was constructed based on the coordinates of the seedlings and saplings. A spatial buffer was applied when extracting canopy characteristics to prevent individuals located at the polygon’s vertices from being influenced by overstory canopy characteristics outside the spatial extent of the local community. In this study, the buffer distance was set to 2.792 m, corresponding to the difference between the radii of the average extended canopy gap (assumed circular) and the average crown opening area (Liu et al., 2013). Redundancy analysis (RDA) was used to assess how canopy environmental features influenced the CWM functional traits of seedling and sapling communities at representative spatial scales.

## Supplemental Information

10.7717/peerj.21819/supp-1 Supplemental Information 1 Leaf morphological and chemical trait characteristics of regenerated seedlings and saplings of potential canopy tree species.

10.7717/peerj.21819/supp-2 Supplemental Information 2 Ecological mechanism spatial pattern diagram of understory regeneration community assembly in broad-leaved Korean pine forest.

10.7717/peerj.21819/supp-3 Supplemental Information 3 Schematic diagram of sampling plot layout.

## Additional Information and Declarations

### Field Study Permissions

The following information was supplied relating to field study approvals (*i.e*., approving body and any reference numbers):

Field experiments were approved by Liangshui National Nature Reserve, The fieldwork certificate has been uploaded as an attachment.

### Data Availability

The following information was supplied regarding data availability:

The data and code are available at Zenodo: DuXinChina. (2025). DuXinChina/Broadleaf.Korean.pine.LAI: Broadleaf.Korean.pine.LAI [Computer software]. Zenodo. https://doi.org/10.5281/zenodo.17015769.

The code is available at Zenodo: DuXinChina. (2025). DuXinChina/M.COMMUNITIES: M.COMMUNITIES [Computer software]. Zenodo. https://doi.org/10.5281/zenodo.17015561.

The data is available at Zenodo: XIN, D. (2025). Survey data of trees and their regeneration, along with leaf trait data. Zenodo. https://doi.org/10.5281/zenodo.16730259.
