# Supplementary material for: Mechanisms of local regeneration and community assembly under broad-leaved Korean pine (Pinus koraiensis) forests in northeastern China
Source: PeerJ. 2026 Nov 4;14:e21819. doi: 10.7717/peerj.21819 (PMC13744168; doi:10.7717/peerj.21819)
Supplement: Supplementary file 1 [file peerj-14-21819-s002.pdf]

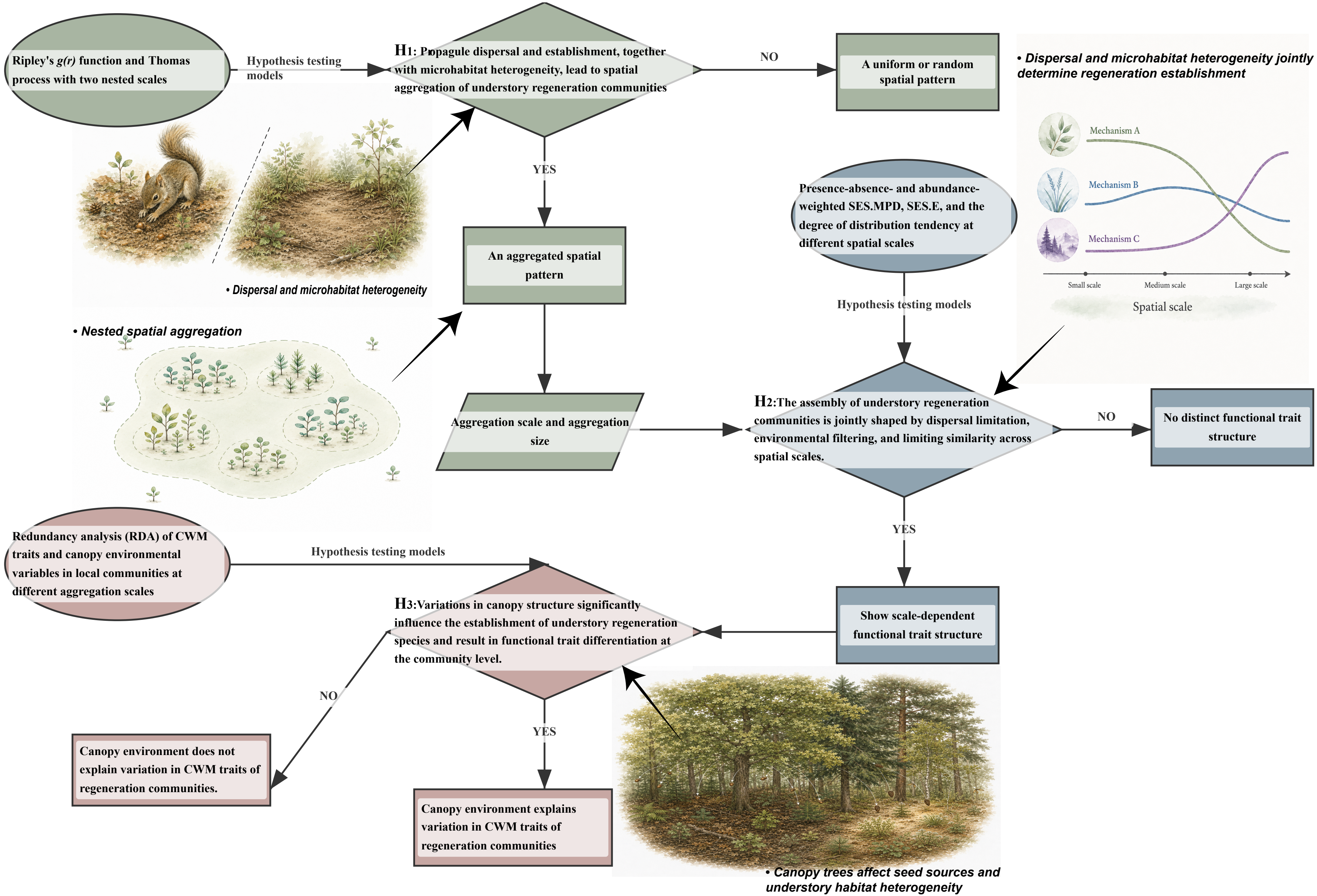
Ripley's g(r) function and Thomas process with two nested scales
Hypothesis testing models
H1: Propagule dispersal and establishment, together with microhabitat heterogeneity, lead to spatial aggregation of understory regeneration communities
NO
A uniform or random spatial pattern
• Dispersal and microhabitat heterogeneity jointly determine regeneration establishment
Mechanism A
Mechanism B
Mechanism C
Small scale
Medium scale
Large scale
Spatial scale
YES
• Dispersal and microhabitat heterogeneity
An aggregated spatial pattern
Presence-absence- and abundance-weighted SES.MPD, SES.E, and the degree of distribution tendency at different spatial scales
Hypothesis testing models
• Nested spatial aggregation
Aggregation scale and aggregation size
H2:The assembly of understory regeneration communities is jointly shaped by dispersal limitation, environmental filtering, and limiting similarity across spatial scales.
NO
No distinct functional trait structure
YES
Redundancy analysis (RDA) of CWM traits and canopy environmental variables in local communities at different aggregation scales
Hypothesis testing models
H3:Variations in canopy structure significantly influence the establishment of understory regeneration species and result in functional trait differentiation at the community level.
Show scale-dependent functional trait structure
NO
YES
Canopy environment does not explain variation in CWM traits of regeneration communities.
Canopy environment explains variation in CWM traits of regeneration communities
• Canopy trees affect seed sources and understory habitat heterogeneity
