# Supplementary material for: Mechanisms of local regeneration and community assembly under broad-leaved Korean pine (Pinus koraiensis) forests in northeastern China
Source: PeerJ. 2026 Nov 4;14:e21819. doi: 10.7717/peerj.21819 (PMC13744168; doi:10.7717/peerj.21819)
Supplement: Supplementary file 2 [file peerj-14-21819-s003.pdf]

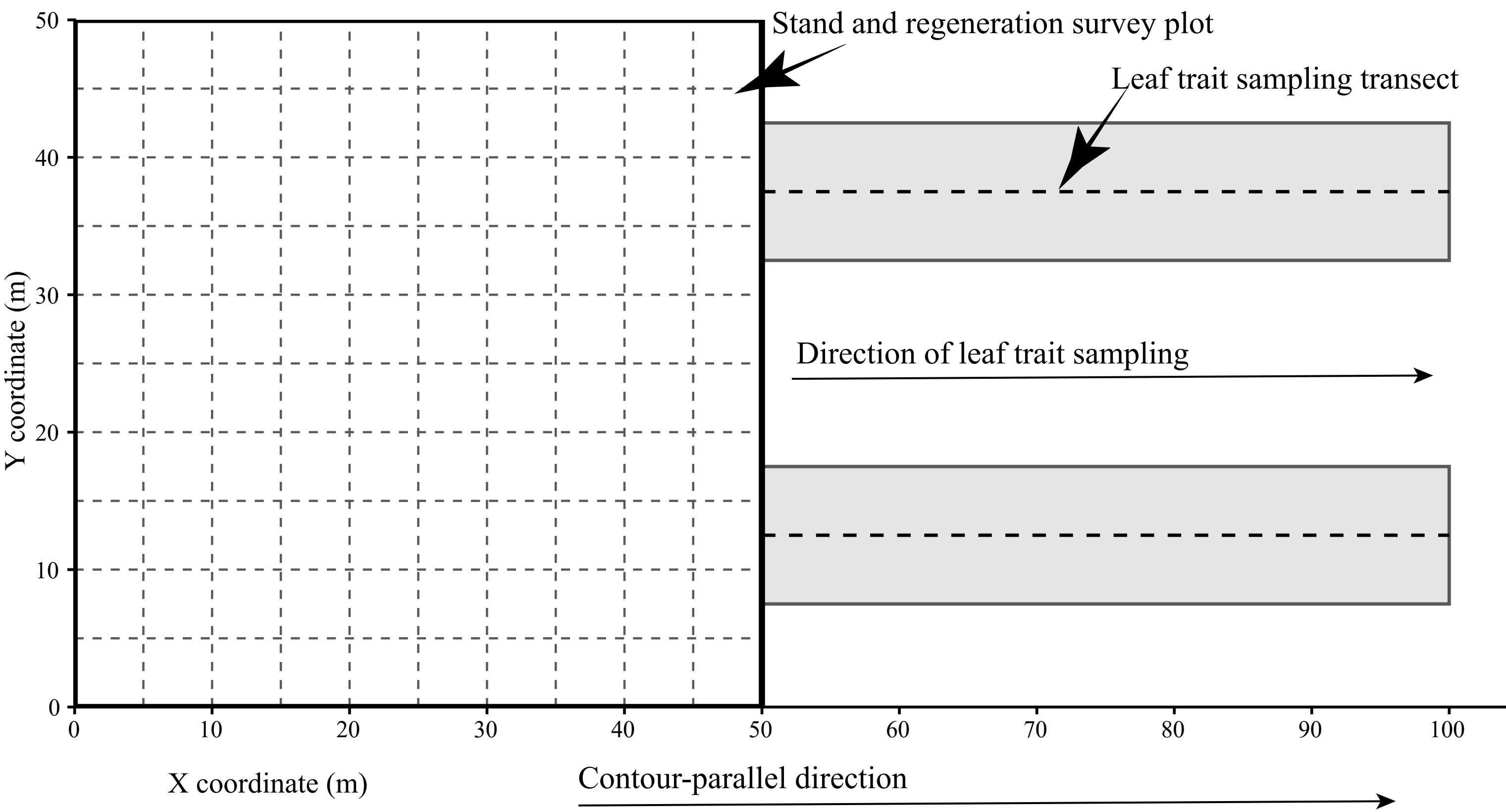
Stand and regeneration survey plot
Leaf trait sampling transect
Direction of leaf trait sampling
Y coordinate (m)
X coordinate (m)
Contour-parallel direction
0
10
20
30
40
50
60
70
80
90
100
