# Supplementary material for: Mechanisms of local regeneration and community assembly under broad-leaved Korean pine (Pinus koraiensis) forests in northeastern China
Source: PeerJ. 2026 Nov 4;14:e21819. doi: 10.7717/peerj.21819 (PMC13744168; doi:10.7717/peerj.21819)
Supplement: Supplementary file 3 [file peerj-14-21819-s001.docx]

**Appendix Table S1.** Leaf morphological and chemical trait characteristics of regenerated seedlings and saplings of potential canopy tree species.

| species | Development stage | C_Mass(mg/g) | N_mass(mg/g) | P_mass(mg/g) | K_mass(mg/g) | N_area(g/m^2^) | P_area(g/m^2^) | C/N | C/P | N/P | LDMC (%) | LL (cm) | LT (mm) | LW (cm) | LA (cm^2^) | SLA  (cm^2^/g) |
| --- | --- | --- | --- | --- | --- | --- | --- | --- | --- | --- | --- | --- | --- | --- | --- | --- |
| *Abies nephrolepis.* | seeding | 534.39 | 22.59 | 1.77 | 6.94 | 2.11 | 0.18 | 23.66 | 301.92 | 12.76 | 0.35(0.03) | 2.37(0.65) | 0.37(0.09) | 0.17(0.02) | 0.38(0.14) | 94.31(17.27) |
| *Abies nephrolepis.* | sapling | 535.14 | 19.26 | 2.06 | 8.46 | 2.34 | 0.21 | 27.79 | 260.03 | 9.36 | 0.35(0.03) | 1.81(0.51) | 0.38(0.14) | 0.15(0.02) | 0.26(0.10) | 85.25(16.76) |
| *Acer momo.* | seeding | 446.26 | 24.60 | 2.04 | 9.86 | 0.97 | 0.08 | 18.14 | 218.75 | 12.06 | 0.34(0.06) | 7.14(1.03) | 0.10(0.02) | 9.67(1.60) | 32.72(8.64) | 251.76(93.22) |
| *Acer momo.* | sapling | 442.98 | 26.85 | 1.52 | 8.66 | 1.08 | 0.06 | 16.50 | 291.43 | 17.66 | 0.39(0.02) | 7.35(1.44) | 0.12(0.02) | 10.37(2.11) | 33.96(13.43) | 249.24(43.36) |
| *Acer tegmentosum.* | seeding | 445.26 | 22.59 | 2.07 | 11.32 | 0.74 | 0.07 | 19.71 | 215.62 | 10.94 | 0.25(0.02) | 13.02(2.86) | 0.11(0.01) | 13.94(3.17) | 122.93(54.93) | 303.42(57.32) |
| *Acer tegmentosum.* | sapling | 450.61 | 27.18 | 1.71 | 9.72 | 1.02 | 0.06 | 16.58 | 263.51 | 15.89 | 0.27(0.03) | 11.79(1.98) | 0.12(0.02) | 11.32(2.04) | 86.99(31.16) | 272.22(51.82) |
| *Acer ukurunduense.* | seeding | 444.35 | 29.45 | 3.58 | 19.18 | 0.93 | 0.11 | 15.09 | 124.05 | 8.22 | 0.21(0.04) | 10.57(2.00) | 0.11(0.02) | 11.20(2.66) | 57.23(21.66) | 316.06(70.67) |
| *Acer ukurunduense.* | sapling | 456.30 | 27.77 | 2.07 | 13.51 | 1.02 | 0.08 | 16.43 | 220.43 | 13.42 | 0.25(0.04) | 10.09(1.62) | 0.15(0.03) | 10.54(2.22) | 56.55(20.87) | 273.05(87.97) |
| *Betula costata.* | seeding | 460.85 | 38.29 | 5.04 | 14.04 | 1.00 | 0.13 | 12.04 | 91.53 | 7.60 | 0.28(0.06) | 7.97(1.39) | 0.09(0.02) | 4.49(0.78) | 21.63(6.66) | 379.70(130.62) |
| *Betula costata.* | sapling | 468.02 | 30.15 | 3.37 | 15.04 | 0.91 | 0.10 | 15.52 | 138.88 | 8.95 | 0.34(0.03) | 7.50(1.16) | 0.09(0.01) | 3.86(0.54) | 16.08(4.15) | 328.15(38.30) |
| *Fraxinus mandshurica.* | seeding | 444.43 | 34.12 | 2.17 | 13.52 | 1.42 | 0.09 | 13.03 | 204.81 | 15.72 | 0.27(0.03) | 6.54(1.41) | 0.12(0.02) | 2.87(0.44) | 12.47(3.95) | 241.37(41.36) |
| *Fraxinus mandshurica.* | sapling | 438.94 | 32.39 | 1.96 | 10.42 | 1.55 | 0.09 | 13.55 | 223.95 | 16.53 | 0.29(0.05) | 7.86(1.12) | 0.1(0.01) | 3.55(0.51) | 18.61(5.21) | 209.1(42.42) |
| *Picea koraiensis.* | seeding | 506.02 | 19.13 | 1.60 | 5.35 | 2.43 | 0.20 | 26.45 | 316.26 | 11.96 | 0.33(0.02) | 1.51(0.39) | 0.38(0.08) | 0.12(0.02) | 0.19(0.06) | 78.09(8.53) |
| *Picea koraiensis.* | sapling | 502.96 | 19.76 | 2.03 | 9.04 | 2.85 | 0.29 | 25.45 | 247.76 | 9.73 | 0.33(0.02) | 1.39(0.34) | 0.54(0.09) | 0.11(0.02) | 0.17(0.06) | 69.51(13.42) |
| *Pinus koraiensis.* | seeding | 503.33 | 18.82 | 1.88 | 7.45 | 3.55 | 0.35 | 26.74 | 268.01 | 10.02 | 0.25(0.03) | 10.34(1.62) | 0.07(0.01) | 0.10(0.01) | 3.00(0.73) | 53.04(7.83) |
| *Pinus koraiensis.* | sapling | 508.21 | 17.16 | 1.62 | 6.71 | 3.68 | 0.35 | 29.62 | 313.71 | 10.59 | 0.30(0.03) | 11.70(14.3) | 0.08(0.01) | 0.10(0.01) | 3.50(0.70) | 45.65(6.17) |
| *Tilia amurensis.* | seeding | 475.36 | 28.96 | 2.22 | 13.08 | 0.85 | 0.07 | 16.41 | 214.51 | 13.07 | 0.31(0.03) | 8.30(1.23) | 0.10(0.01) | 7.61(0.98) | 41.64(11.97) | 341.66(41.15) |
| *Tilia amurensis.* | sapling | 471.25 | 28.13 | 2.73 | 11.76 | 1.00 | 0.10 | 16.75 | 172.62 | 10.30 | 0.34(0.04) | 7.93(1.35) | 0.13(0.01) | 7.38(1.29) | 38.90(13.80) | 281.89(37.26) |
| *Ulmus japonica.* | seeding | 426.52 | 26.78 | 2.31 | 15.83 | 1.03 | 0.09 | 15.93 | 184.56 | 11.59 | 0.29(0.05) | 7.27(1.76) | 0.10(0.01) | 4.13(0.88) | 16.49(7.49) | 265.26(51.70) |
| *Ulmus japonica.* | sapling | 417.66 | 21.66 | 3.35 | 12.93 | 1.17 | 0.18 | 19.28 | 124.67 | 6.47 | 0.39(0.03) | 8.25(1.68) | 0.14(0.02) | 4.34(0.88) | 20.08(7.34) | 184.46(42.28) |
| *Ulmus laciniata.* | seeding | 395.88 | 29.74 | 2.04 | 17.51 | 1.00 | 0.07 | 13.31 | 194.44 | 14.61 | 0.28(0.04) | 10.55(2.11) | 0.12(0.03) | 6.92(1.45) | 39.03(14.04) | 298.86(56.67) |
| *Ulmus laciniata.* | sapling | 381.81 | 24.74 | 1.99 | 15.41 | 1.07 | 0.09 | 15.43 | 191.86 | 12.43 | 0.35(0.02) | 11.47(3.27) | 0.14(0.01) | 7.16(2.10) | 50.62(30.35) | 231.04(37.23) |
